# Selective Nitridation Crafted a High‐Density, Carbon‐Free Heterostructure Host with Built‐In Electric Field for Enhanced Energy Density Li–S Batteries

**DOI:** 10.1002/advs.202201823

**Published:** 2022-06-16

**Authors:** Hongmei Wang, Yunhong Wei, Guochuan Wang, Yiran Pu, Li Yuan, Can Liu, Qian Wang, Yun Zhang, Hao Wu

**Affiliations:** ^1^ Engineering Research Center of Alternative Energy Materials & Devices Ministry of Education College of Materials Science and Engineering Sichuan University Chengdu Sichuan 610065 China; ^2^ Hefei National Laboratory for Physical Sciences at the Microscale Department of Applied Chemistry University of Science and Technology of China Hefei 230026 China

**Keywords:** built‐in electric field, heterostructures, high tap density, Li–S full batteries, volumetric energy density

## Abstract

To achieve both high gravimetric and volumetric energy densities of lithium–sulfur (Li–S) batteries, it is essential yet challenging to develop low‐porosity dense electrodes along with diminishment of the electrolyte and other lightweight inactive components. Herein, a compact TiO_2_@VN heterostructure with high true density (5.01 g cm^–3^) is proposed crafted by ingenious selective nitridation, serving as carbon‐free dual‐capable hosts for both sulfur and lithium. As a heavy S host, the interface‐engineered heterostructure integrates adsorptive TiO_2_ with high conductive VN and concurrently yields a built‐in electric field for charge‐redistribution at the TiO_2_/VN interfaces with enlarged active locations for trapping‐migration‐conversion of polysulfides. Thus‐fabricated TiO_2_@VN–S composite harnessing high tap‐density favors constructing dense cathodes (≈1.7 g cm^–3^) with low porosity (<30 vol%), exhibiting dual‐boosted cathode‐level peak volumetric‐/gravimetric‐energy‐densities nearly 1700 Wh L^−1^_cathode_/1000 Wh kg^−1^_cathode_ at sulfur loading of 4.2 mg cm^−2^ and prominent areal capacity (6.7 mAh cm^−2^) at 7.6 mg cm^−2^ with reduced electrolyte (<10 µL mg^−1^_sulfur_). Particular lithiophilicity of the TiO_2_@VN is demonstrated as Li host to uniformly tune Li nucleation with restrained dendrite growth, consequently bestowing the assembled full‐cell with high electrode‐level volumetric/gravimetric‐energy‐density beyond 950 Wh L^−1^_cathode+anode_/560 Wh kg^−1^_cathode+anode_ at 3.6 mg cm^−2^ sulfur loading alongside limited lithium excess (≈50%).

## Graphical abstract

A high‐density fibrous TiO_2_@VN heterostructure mediator with built‐in electric fields is constructed via an ingenious selective nitridation from biotemplate‐derived twinborn hybrid titanium/vanadium oxide fibers, which can synchronously serve as carbon‐free, dual‐capable hosts of both sulfur and lithium for practical high‐energy‐density Li–S full batteries.

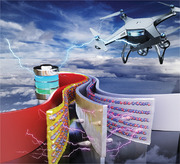

## 1 Introduction

Exploring desirable energy density storage systems has provoked keen interests to develop portable electric devices and electronic vehicles.^[^ ^1^, ^2^ ^]^ Among all the secondary batteries, lithium–sulfur (Li–S) battery is deemed to be one of the most promising system due to its extremely high theoretical gravimetric energy density (*E*_G_, 2600 Wh kg^−1^), satisfactory volumetric energy density (*E*_V_, 2800 Wh L^−1^), as well as environmental benignity.^[^ ^3^, ^4^, ^5^ ^]^ However, the practical application of Li–S batteries is still restricted by the inevitable obstacles, such as poor electrical conductivity of S and its discharge products (Li_2_S_2_/Li_2_S), large volume expansion, and shuttle effect of soluble polysulfides (LiPS).^[^ ^6^ ^]^ To address these problems, tremendous strategies have been proposed. For instance, porous carbon materials can provide sulfur cathode with a conductive network and physical confinement of LiPS, but the dissolution and shuttle effect of LiPS cannot be effectively suppressed.^[^ ^7^, ^8^, ^9^, ^10^, ^11^ ^]^ Additionally, polar metal compounds, such as metal oxides and sulfides are considered to be alternatives to alleviate the shuttle effect by forming strong chemical bonds with LiPS.^[^ ^12^, ^13^ ^]^ Nonetheless, the poor electrical conductivity of metal oxides and sulfides makes it hard to promote the conversion kinetic of LiPS and deposition behavior of the Li_2_S. Comparatively, metal nitrides and carbides exhibit higher conductivity (e. g. 1.23 × 10^4^ S cm^−1^ for VN,^[^ ^14^, ^15^ ^]^ 4.55 × 10^4^ S cm^−1^ for MoN^[^ ^16^ ^]^ and 1.41 × 10^4^ S cm^−1^ for Mo_2_C^[^ ^17^ ^]^) but weaker adsorption ability than oxides. Hence, it is not easy to identify an exclusive material that can simultaneously satisfy the requirements of high conductivity to promote electron/ion transport, targeted adsorption ability to immobilize LiPS, and catalytic action to accelerate the LiPS conversion.

Recently, engineering of heterostructured S host has been put forward in Li–S batteries, since there are distinct phase interfaces between heterocrystals with different bandgaps that can provide synergistic rapid electron conduction with good chemisorption. Amongst the reported heterostructures, an oxide‐nitride heterojunction has been proved as a desired candidate for high‐performance Li–S batteries. For example, with a graphene‐protected TiN–TiO_2_ heterostructure, the Li–S battery achieves favorable cycling stability with a low decay of 0.054% per cycle within 1000 cycles at 2 C, attributed to the synergistic effect of high catalytic and LiPS adsorption abilities from TiN and TiO_2_, respectively.^[^ ^18^ ^]^ As to the construction of such oxide‐nitride heterostructures, the most popular approach is the ammonia annealing treatment of a monometallic oxide precursor, partially transforming it into the nitride component by manipulating the ammonization temperature or time, such as TiO_2_ for TiO_2_–TiN,^[^ ^19^ ^]^ MoO_3_ for MoO_2_‐MoN^[^ ^20^ ^]^ and Nb_2_O_5_ for Nb_2_O_5_–Nb_4_N_5_.^[^ ^21^ ^]^ To our surprise, however, little attention has been paid to the preparation of nonhomogeneous metal‐based oxide‐nitride heterostructures, while still the affection of such kinds of heterostructures in the Li–S electrochemistry remains uninvestigated. Ti and V are well‐known low‐cost elements with abundant reserve. When applied as the sulfur host in Li–S batteries, TiO_2_ has been proved with strong chemical adsorption properties for immobilizing polysulfide species,^[^ ^22^, ^23^, ^24^ ^]^ while VN with high electronic conductivity is able to facilitate rapid electron transfer to the adsorption sites of polysulfides.^[^ ^9^, ^25^, ^26^ ^]^ Thus, combining these two components into a heterostructure could be one of the viable and effective strategies for synergistically tuning S electrochemistry behaviors toward high‐performance Li–S batteries.

On the other hand, the vast majority of reported heterostructure‐based S cathodes have focused only on gravimetric capacity and cycling life, yet the concern on volumetric capacity and corresponding *E*_V_ has gone unheeded. Besides, to improve the utilization of active materials, the heterostructures are usually supported on or coupled with various carbon nanomaterials. These lightweight inactive carbon matrix with large surface area and excess void spaces generally account for nearly 30 wt% of the whole cathode, which inevitably lead to poor scalability, low tap density (<1.0 g cm^−3^), high porosity (>60 vol%), excessive electrolytes (>15 µL mg^−1^_sulfur_), and inferior volumetric capacity of the resultant S‐cathodes.

Generally speaking, the *E*_V_ is one of the substantial requirements as significant as the *E*_G_ and cycling life for practical high‐energy secondary batteries. Particularly, for a portable electronic device, it is crucial to provide sufficient capacity in a limited space. For enhancing the *E*_V_ of Li–S batteries, two broad categories of strategies have been attempted in recent years. One is enhancing the content of active sulfur and the other is improving the density of S cathode. Increasing sulfur content is not conducive to the efficient utilization of active substances. In consequence, introducing the carbon‐free heavy hosts with high density has drawn considerable attention, such as NiCo_2_O_4_,^[^ ^27^ ^]^ MnO_2_,^[^ ^28^ ^]^ CoOOH,^[^ ^29^ ^]^ CeO_2_,^[^ ^30^ ^]^ CoMn_2_O_4_,^[^ ^31^ ^]^ Ti_3_C_2_T*_x_*,^[^ ^32^ ^]^ RuO_2_,^[^ ^33^ ^]^ Ti_3_C_2_@iCON^[^ ^34^ ^]^ and La_0.8_Sr_0.2_MnO_3_.^[^ ^35^ ^]^ Specifically, S/CoOOH composites with a tap density of 1.26 g cm^−3^ were reported to deliver a high *E*_V_ of 1493 Wh L^−1^_cathode_ under the sulfur loading of 4.35 mg cm^−2^.^[^ ^29^ ^]^ Despite these advances, there are very few literatures regarding the attempts of carbon‐free heterostructure hosts for simultaneous regulation of both S cathode and Li anode, not to mention the realization of high *E*_V_ in working Li–S full batteries.

Taking the above into consideration, here we proposed an effective strategy toward TiO_2_@VN heterostructure engineering via an ingenious selective nitridation from biotemplate‐derived twinborn titanium/vanadium oxide hybrid fibers_._ The resulted carbon‐free, compact TiO_2_@VN heterostructure exhibits moderate surface area (28.8 m^2^ g^−1^), high true density (5.01 g cm^−3^), and strong amphiphilicity, which promise it as an ideal dual‐capable heavy host to construct dense S and Li electrodes, targeting high‐energy‐density Li–S full batteries. As a heavy S host, the heterostructured TiO_2_@VN fiber is favorable for constructing a high‐tap‐density S‐composite (1.52 g cm^−3^) and densely compacted cathode. More importantly, the quasi‐metallic VN can spontaneously induce a built‐in electric field pointing toward the semiconducting TiO_2_ at the interfaces so as to drive charge transfer from VN to TiO_2_. Such specific interfacial characteristics of TiO_2_@VN not only remarkably strengthen the adsorption and spatial distribution of LPS, but also allow their consistent catalytic conversion along with reduced Li_2_S precipitation/decomposition barrier. As a result, the S‐loaded TiO_2_@VN composite (denoted as TiO_2_@VN‐S) exhibits a superb rate capability of 594 mAh g^−1^ at 3 C and excellent cycling performance with a low decay rate of 0.054% per cycle after 500 cycles. Besides, dense TiO_2_@VN–S cathodes with a desirable cathode density (1.69 g cm^−3^) and porosity (28 vol%) attain dual‐enhanced cathode‐level peak volumetric/gravimetric energy densities of nearly 1650 Wh L^−1^_cathode_/1000 Wh kg^−1^_cathode_ at sulfur loading of 4.2 mg cm^−2^ and low electrolyte‐to‐sulfur (E/S) ratio (7 µL mg^−1^_sulfur_), together with an admirable areal capacity of 6.7 mAh cm^−2^ at an elevated loading of 7.6 mg cm^−2^. When served as the Li metal host, the TiO_2_@VN heterostructure with specific lithiophilicity is also beneficial for controlling Li deposition and supporting Li nucleation, thus leading to highly reversible, dendrite‐free Li metal electrodes. Combining these merits, the consequent Li–S full batteries constructed with the TiO_2_@VN as both cathode and anode hosts is able to deliver considerable electrode‐level *E*_V_/*E*_G_ of 951 Wh L^−1^_cathode+anode_/562 Wh kg^−1^_cathode+anode_ and good cycling over 150 cycles even at high sulfur loading (3.6 mg cm^−2^) with controlled low negative/positive capacity ratio (N/P: ≈1.5).

## 2 Results and Discussion

**Figure 1:**
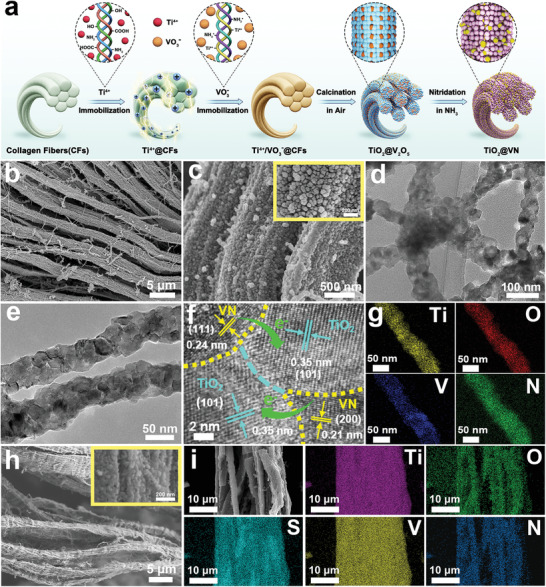
a) Schematic illustration of the synthesis procedure of fibrous TiO_2_@VN heterostructure. b,c) FESEM images, d,e) TEM images, f) HRTEM images, and g) EDX elemental mappings of TiO_2_@VN heterostructure. h) FESEM images and (i) EDX elemental mappings of the TiO_2_@VN–S composite.

The schematic in **Figure** **1**a elucidates the synthesis procedure of fibrous TiO_2_@VN heterostructure based on the employment of natural collagen fibers (CFs) as biotemplate. CFs possess multiple functional groups (—OH, —COOH, and —NH_2_) that are prone to complex with diverse metal cations (e.g., Ti^4+^, Zr^4+^, Ni^2+^, and Mg^2+^).^[^ ^19^ ^]^ Thus, Ti^4+^ ions are first coordinated with the —OH and —COOH on the CFs, forming Ti^4+^ modified CFs (denoted as Ti^4+^@CFs). After that, the surface of Ti^4+^@CFs is positively charged at a low pH value (Figure S1, Supporting Information), which could facilitate the electrostatic adherence of negatively charged VO_3_^−^ with Ti^4+^@CFs (denoted as Ti^4+^/VO_3_^−^@CFs). Besides, when immersed in an acid solution, the —NH_2_ on the CF chains becomes positive, which also can react with the negatively charged VO_3_^−^ by electrostatic interaction. Moreover, the special supramolecular fibrous structure of CFs is regarded as an ideal template for preparing fiber‐shaped inorganic superstructures. As such, the subsequent direct calcination treatment of Ti^4+^/VO_3_^−^@CFs in the air atmosphere can lead to the complete decomposition of CFs template, meanwhile leaving behind a fibrous twinborn titanium/vanadium oxide framework, i.e., TiO_2_@V_2_O_5_, as validated by X‐ray diffraction (XRD) patterns (Figure S2, Supporting Information) and field‐emission scanning electron microscopy (FESEM) images (Figure S3, Supporting Information). According to the strength of chemical bonds, the dissociation energy of the Ti—O bond is ≈672.4 kJ mol^−1^, much higher than that of the V—O bond (≈626.8 kJ mol^−1^),^[^ ^36^ ^]^ whilst the Gibbs formation energy of the Ti—N bond (−243.8 kJ mol^−1^) is more negative than that of the V—N bond (−191.1 kJ mol^−1^).^[^ ^37^ ^]^ This means that by annealing at a relatively low temperature in the NH_3_ atmosphere (e.g., 600°C in our work), we can realize a selective nitridation of TiO_2_@V_2_O_5_ so as to convert V_2_O_5_ into VN while retaining TiO_2_ invariable, i.e., TiO_2_@VN heterostructure, as illustrated in Figure 1a. For comparison, the other two kinds of fibrous single‐component sample, TiO_2_ and VN, were also prepared individually by a similar process, in which the CFs was cross‐linked by the monometallic salt (Ti^4+^ or VO_3_^−^). The FESEM images showing the fibrous morphologies of TiO_2_ and VN samples are displayed in Figure S4 (Supporting Information).

The structural characterization of the as‐prepared TiO_2_@VN was investigated by FESEM and transmission electron microscopy (TEM). As exhibited in Figure 1b,c, the TiO_2_@VN shows a well‐maintained fibrous structure with an external diameter of 2–5 µm, which is constituted by nano‐scale fibrils of ≈50–100 nm. Each fibril is formed by the accumulation of nanoparticles with a loose and rough surface, which can be attributed to the removal of CFs template during oxidizing pyrolysis. The TEM images in Figure 1d,e confirm the interconnected fibrous structure of TiO_2_@VN composed of irregular nanoparticles with a size of around 50 nm. The high‐resolution TEM (HRTEM) image in Figure 1f exhibits the well‐defined lattice fringes with spacings of 0.35, 0.21, and 0.24 nm, corresponding to the dominant exposed (101) plane of anatase TiO_2_, the (111) and (200) planes of VN, respectively. More notably, Figure 1f clearly reveals the TiO_2_@VN heterojunction, showing the multiple heterointerfaces of TiO_2_ and VN crystals, such as TiO_2_ (101)/VN (111) and TiO_2_ (101)/VN (200).^[^ ^38^, ^39^, ^40^ ^]^ High‐angle annular dark‐field scanning TEM (HAADF–STEM) combined with energy‐dispersive X‐ray (EDX) elemental mapping verifies the homogeneous distribution of Ti, V, O, and N elements in the fibrous TiO_2_@VN heterostructure (Figure 1g). Figure 1h displays the FESEM images of TiO_2_@VN–S composite at different magnifications. It is clear to see that after the S loading, the fibrous morphology of TiO_2_@VN is still maintained well. Moreover, as illustrated by EDX mapping, there exists uniform spatial distribution of Ti, V, N, O, and S elements in the TiO_2_@VN–S. Notably, the S element is almost overlapped with those of host elements (Figure 1i), implying that S is highly distributed in the TiO_2_@VN heterostructure host by the conventional melt‐diffusion treatment. The corresponding FESEM images and EDX mapping of control samples, TiO_2_–S and VN–S, are also obtained and presented in Figure S5 (Supporting Information). Thermogravimetric analysis (TGA) reveals that the fibrous TiO_2_@VN heterostructure as sulfur host is able to attain typical sulfur loading as high as 69.5 wt% (Figure S6, Supporting Information). Besides, all the characteristic X‐ray diffraction (XRD) peaks corresponding to crystalline S (JCPDS No.08–0247) can be detected in resulted sulfur‐loaded composites, i.e., TiO_2_@VN–S, TiO_2_–S, and VN–S, as depicted in Figure S7 (Supporting Information).

**Figure 2:**
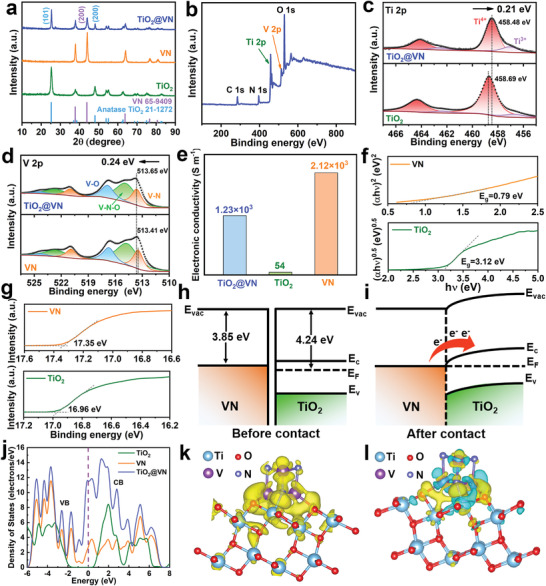
a) XRD patterns of TiO_2_@VN, TiO_2_, and VN. b) XPS survey spectra and high‐resolution XPS spectra of c) Ti 2p and d) V 2p. (e) Electrical conductivity of TiO_2_@VN, TiO_2_, and VN. f) Tauc's bandgap plots and g) UPS spectra of TiO_2_ and VN. Schematic energy bands diagrams of h) VN and TiO_2_ before and i) after contact. (j) DOS profiles of TiO_2_ and TiO_2_@VN. k) Partial charge density around Fermi level of 0.05 eV, the iso‐surface value of yellow contour is 0.00015 e bohr^−3^. l) Differential charge density between TiO_2_ and VN. The cyan and yellow contour indicates reduced and augmented charge density, respectively.

The crystal phase composition of TiO_2_@VN heterostructure was analyzed by XRD measurement. As shown in **Figure** **2**a, TiO_2_@VN shows a series of characteristic diffraction peaks in accordance with anatase TiO_2_ (JCPDS No.21‐1272) and VN (JCPDS No.65‐9409) crystal phases. Consistent XRD patterns were also obtained for control samples, TiO_2_ and VN. It should be noted that the TiO_2_ counterpart shows the identical anatase crystal structure to the TiO_2_@VN heterostructure, further confirming that the lower ammonization temperature (600 °C) adopted in our work is not enough to convert the TiO_2_ into TiN. To characterize the surface chemistry of TiO_2_@VN heterostructure, X‐ray photoelectron spectroscopy (XPS) was conducted. As illustrated in Figure 2b, the XPS survey spectrum confirms the co‐existence of Ti, V, N, O elements in the TiO_2_@VN heterostructure. For the high‐resolution Ti 2p spectrum of TiO_2_@VN (the up half of Figure 2c), two peaks at binding energies of 458.48 (Ti 2p_3/2_) and 464.12 eV (Ti 2p_1/2_) are ascribed to Ti^4+^, while two additional small peaks attributed to the Ti^3+^ can be also observed at 457.08 and 462.61 eV. In addition, the O 1s spectrum (Figure S8a, Supporting Information) displays the presence of oxygen vacancies located at 531.1 eV in TiO_2_@VN heterostructure. This indicates the presence of a small amount of oxygen vacancies in the TiO_2_ species, which is attributed to the ammonia annealing treatment. This can be also verified by the appearance of low‐valent Ti^3+^ XPS peaks in Ti 2p spectrum and the oxygen vacancy peak in O 1s spectrum of TiO_2_ counterpart (the bottom half of Figure 2c and Figure S8b (Supporting Information). As shown in Figure S9 (Supporting Information), electron paramagnetic resonance (EPR) measurement was also performed and a g value of 2.003 corresponding to oxygen defects from excess unpaired electrons can be detected, further verifying the existence of oxygen vacancies in TiO_2_ and TiO_2_@VN. Figure 2d (the up half) exhibits the deconvolution of the V 2p_3/2_ spectrum, where the peaks at 513.65, 514.86, and 516.85 eV can be assigned to the V–N (V^3+^), V–N–O (V^4+^), and V–O (V^5+^), respectively. The appearance of V—N—O and V—O bonds are mainly ascribed to the inevitable oxidation of the air‐exposed VN nanoparticles during the XPS test.^[^ ^41^ ^]^ The relative mass fraction of TiO_2_ and VN in this heterostructure was estimated to be about 67 and 33 wt%, according to the results measured by inductively coupled plasma optical emission spectroscopy (ICP–OES) (Table S1, Supporting Information). The N_2_ adsorption/desorption isotherms and pore‐size distribution of TiO_2_@VN heterostructure are displayed in Figure S10 (Supporting Information). A typical IV isotherm with a hysteresis loop in TiO_2_@VN indicates the existence of a hierarchical micro‐mesoporous structure. Based on the Brunauer‐Emmett‐Teller (BET) calculation method, the specific surface area of TiO_2_@VN heterostructure is estimated to be 28.8 m^2^ g^−1^. For comparison, the BET specific surface area of TiO_2_ and VN samples are measured as 12.6 and 57.1 m^2^ g^−1^, respectively. Notably, compared with the single‐component TiO_2_, there exists a significant increase in the specific surface area of TiO_2_@VN as a result of the formation of VN. The enlarged specific surface area is favorable to the distribution of sulfur as well as the electrolyte permeation.

Figure 2e displays the measured electrical conductivities of samples. Obviously, the conductivity of TiO_2_@VN (1.23×10^3^ S m^−1^) shows the same order of magnitude as that of VN (2.12×10^3^ S m^−1^), but nearly 23–folds higher than that of TiO_2_ (54 S m^−1^). This indicates that the enhanced conductivity of TiO_2_@VN is associated with the involvement of quasi‐metallic VN, which is also verified by the density of states (DOS) calculations conducted from density functional theory (DFT). As shown in Figure 2j, the band gap for the TiO_2_ between the valance band (VB) and the conduction band (CB) is ≈3.0 eV. By contrast, the incorporated VN has metallic characteristics, and thus it is capable of posing visible contributions on the DOS near the Fermi level, which lead to a higher dispersion of band structure in the TiO_2_@VN together with enhanced electroconductivity. To further verify the contribution of each element to the density of states of TiO_2_@VN heterostructure, the partial density of states (PDOS) of TiO_2_@VN heterostructure was calculated. As shown in Figure S11 (Supporting Information), the density of states near Fermi level of TiO_2_@VN heterostructure are principally contributed by VN, while the contribution of TiO_2_ is mainly concentrated on its own conduction band and valence band. In addition, it can be found that the contribution of V is much higher than that of N. Given the actual content of VN in our prepared TiO_2_@VN heterostructure is less than 35 wt%, it is reasonable that the measured electrical conductivity of TiO_2_@VN is slightly below that of VN (Figure 2e). Ultraviolet–visible diffuse reflectance spectra (UV–vis DRS) were also carried out to estimate the bandgap values of the VN and TiO_2_ (Figure S12, Supporting Information). According to the Tauc plots based on Kubelka–Munk equation, as shown in Figure 2f, the bandgap values of VN and TiO_2_ are 0.79 and 3.12 eV, respectively. This agrees well with the above calculation and experiment results. To further uncover the synergistic effects of the quasi‐metallic (VN) and semiconducting (TiO_2_) phases in the as‐designed heterostructure, ultraviolet photoelectron spectroscopy (UPS) technique was performed to investigate the work function of the VN and TiO_2_. As shown in Figure 2g, the work function value of VN is calculated to be 3.85 eV (the calculation details are described in Supporting Information), much less than that of the TiO_2_ (4.24 eV). The calculated work function values of VN and TiO_2_ in our work are fairly close to those reported in literatures.^[^ ^42^, ^43^, ^44^, ^45^ ^]^ The difference in the work function suggests that there would be band bending and self‐driven electron transfer from VN to TiO_2_ at their coupling interfaces during contact until the work function equilibrium is reached. According to the schematic energy band diagrams illustrated in Figure 2i,h, a spontaneous built‐in electric field pointing toward the TiO_2_ side can be formed when the quasi‐metallic VN and semiconducting TiO_2_ are in contact. Thus‐generated built‐in electric field could favor a spatially optimized distribution of active LPS species since the polysulfide anions (e.g., S_6_^2−^, S_4_^2−^) once adsorbed on the TiO_2_ can be directionally migrated to the positively charged VN side. Meanwhile, the induced charge redistribution at the interfaces is also expected to expedite the electron transport and Li‐ion diffusion between VN and TiO_2_, thereby enlarging the electroactive locations for catalytic conversion of polysulfides. The partial charge density analysis (Figure 2k) based on DFT further confirms that the states of TiO_2_@VN around the Fermi level are principally contributed by the VN. Besides, the differential charge density plot in Figure 2l demonstrates that an obvious charge transfer from VN to TiO_2_ can occur at the heterointerface, which is consistent with the UPS results. Similar results can be also identified from the XPS analysis (Figure 2c,d). Compared with the Ti 2p spectrum of TiO_2_, the Ti—O bond location of TiO_2_@VN shows an obvious blue‐shift of ≈0.21 eV (Figure 2c), while a red‐shift of ≈0.24 eV is observed for the V—N bond in the V 2p spectra of TiO_2_@VN relative to that of VN (Figure 2d), indicating the electron transfer from VN to TiO_2_ at the interfaces of TiO_2_@VN heterostructure.

**Figure 3:**
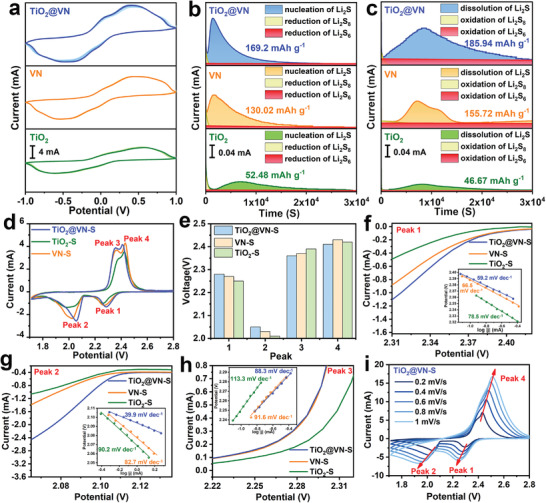
a) The first five CV curves of symmetrical cell of TiO_2_@VN, VN, and TiO_2_ at 3 mV s^−1^ in electrolyte with Li_2_S_6_. Chronoamperometry curves at b) 2.05 V and c) 2.40 V to evaluate the nucleation and decomposition kinetics of Li_2_S on TiO_2_@VN, VN, and TiO_2_ electrodes with Li_2_S_8_/tetraglyme solution. d) CV curves of the TiO_2_@VN–S, TiO_2_–S, and VN–S electrodes at the scan rate of 0.1 mV s^−1^. e) The comparison of peak voltages for TiO_2_@VN–S, TiO_2_–S, and VN–S electrodes from the CV curves in (d). Tafel plots of f) the first cathodic reduction process, g) the second cathodic reduction process, and h) the first anodic oxidation process. i) CV curves of TiO_2_@VN–S at different scan rates.

To evaluate the electrocatalytic activity of TiO_2_@VN, VN, and TiO_2_, symmetric cells were assembled with two identical electrode materials. As exhibited in **Figure** **3**a, the cyclic voltammetry (CV) curves of three samples over the five cycles are well‐overlapped, demonstrating the good reversibility of TiO_2_@VN, VN, and TiO_2_ electrodes. By comparison, the presence of VN renders a larger current density for the TiO_2_@VN electrode over the TiO_2_ electrode, indicating that the VN component can substantially promote the lithiation‐delithiation reaction kinetics of LiPS conversion. The symmetric battery with the TiO_2_@VN electrode in the absence of Li_2_S_6_ shows a negligible current, which suggests Li_2_S_6_ is the only active specie in the redox reaction system (Figure S13, Supporting Information). More importantly, when the scan rate increases from 3.0 to 9.0 mV s^−1^, the redox peaks of the symmetric cells with TiO_2_@VN and VN electrodes can still be observed clearly, reflecting the redox kinetics promoted by TiO_2_@VN and VN better than that by TiO_2_ (Figure S14, Supporting Information). To further investigate the impact of the TiO_2_@VN heterostructure on the liquid‐solid conversion, Li_2_S nucleation and decomposition experiments were performed by pairing TiO_2_@VN cathode and Li foil anode with the Li_2_S_8_/tetraglyme catholyte. For the nucleation of Li_2_S (Figure 3b), when discharging at the constant potential of 2.05 V, both TiO_2_@VN and VN electrodes possess much shorter nucleation time and higher peak currents (0.14 mA at 980s and 0.11 mA at 1070s, respectively) compared with the TiO_2_ electrode (0.035 mA at 2918s), suggesting the higher catalytic efficiency on TiO_2_@VN and VN electrodes towards Li_2_S nucleation. Beyond that, the Li_2_S nucleation capacities on TiO_2_@VN and VN electrodes are calculated to be 169.2 and 130.02 mAh g^−1^, respectively, much higher than that on the TiO_2_ electrode (52.48 mAh g^−1^). These results imply that the VN component in TiO_2_@VN heterostructure can provide more active sites to realize the high capacity of the Li_2_S precipitation. For the decomposition of Li_2_S (Figure 3c), the potentiostatic charge curve of the TiO_2_@VN electrode also exhibits a higher current response than that of the TiO_2_ electrode, indicating a lower oxidation overpotential for Li_2_S dissolution. Moreover, the increased dissolution capacity of TiO_2_@VN and VN, calculated as 185.94 and 155.72 mAh g^−1^, respectively, can be also obtained, suggesting that the boosted conversion efficiency from solid Li_2_S to liquid LiPS could be largely attributed to the existence of VN.

Encouraged by the above virtues, CV tests were carried out from 1.7 to 2.8 V (vs Li/Li^+^) to explore the electrochemical behavior of the sulfur redox kinetics on the TiO_2_@VN–S, TiO_2_–S, and VN–S. Figure 3d gives the typical CV curves for all the prepared electrodes at a scanning rate of 0.1 mV s^−1^, in which two pairs of redox peaks are associated with the reversible electrochemical reaction between solid S_8_ and soluble LiPS (peak 1/peak 4) and the conversion between soluble LiPS and insoluble Li_2_S (peak 2 and peak 3) can be observed.^[^ ^41^ ^]^ Compared with the TiO_2_–S and VN–S, the TiO_2_@VN–S shows more positive peak potentials for the two reduction peaks (peak 1 and peak 2) while the peak potentials for its two oxidation peaks (peak 3 and peak 4) seem more negative (Figure 3e), demonstrating the lower polarization stimulated by the TiO_2_@VN heterostructure. The CV curves of TiO_2_@VN–S and control samples are differentiated to obtain the onset potentials of two reduction peaks (peak 1 and 2) and one oxidation peak (peak 3) (Figure S15, Supporting Information). Compared to the TiO_2_–S electrode, the VN–S and TiO_2_@VN–S ones show relatively larger reduction onset potentials but smaller oxidation onset potentials. To better understand the catalytic activity of TiO_2_@VN heterostructure, we compare the Tafel plots of two cathodic cycles and one anodic cycle, as shown in Figure 3f–h. For the two reduction processes, the Tafel slopes of the TiO_2_@VN–S electrode are estimated to be 59.2 mV dec^−1^ (peak 1) and 39.9 mV dec^–1^ (peak 2), obviously smaller than those of VN–S and TiO_2_–S ones (66.5/78.5 and 82.7/90.2 mV dec^−1^, respectively). Likewise, as for the oxidation process, the TiO_2_@VN–S also displays a much smaller line gradient of 88.3 mV dec^−1^ in comparison with the VN–S and TiO_2_–S (91.6 and 113.3 mV dec^−1^, respectively), demonstrating the enhanced redox conversion between LiPS and Li_2_S on the TiO_2_@VN–S electrode. Considering the relatively low content of VN (≈33 wt%) in the TiO_2_@VN, it suggests that there could be a synergistic effect of VN and TiO_2_ associated with the spontaneous built‐in electric field in such a designed heterostructure on modulating the sulfur redox behavior. The Li‐ion diffusion behaviors were conducted using CV tests under a series of scan rates from 0.2 to 1 mV s^−1^. As expected in Figure 3i and Figures S16–S17 (Supporting Information), all the peak currents show a good linear relationship with the square root of scanning rates, signifying a diffusion‐controlled redox behavior of sulfur cathodes. The Li‐ion diffusion coefficients (*D*_Li_^+^) were calculated by the Randles‐Sevcik equation, as shown in Table S2 (Supporting Information), in which the *D*_Li_^+^ either in the reduction or oxidation process feature the maximum values based on the binary TiO_2_@VN heterogeneous over the two other single‐component samples. The enhanced ion diffusion in TiO_2_@VN–S could be credited to the structure, which can provide stable interface contact for adsorbing and transferring of LiPS.^[^ ^46^, ^47^, ^48^ ^]^

**Figure 4:**
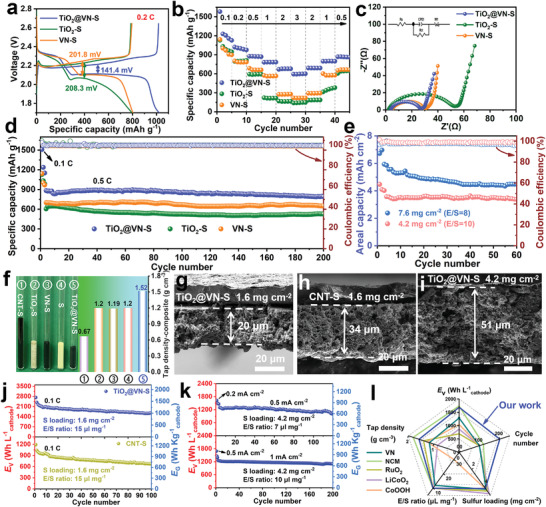
a) Typical galvanostatic charge‐discharge profiles, b) rate capability, c) Nyquist plots, and d) cycling performance of TiO_2_@VN–S, TiO_2_–S, and VN–S cathodes. e) Cycling performance of TiO_2_@VN–S cathode with raised sulfur loading and lean electrolyte. f) Comparison of different samples with equal quality and their tap density: CNT–S, TiO_2_–S, VN–S, pure S, and TiO_2_@VN–S. g–i) Cross‐section FESEM images of CNT–S and TiO_2_@VN–S electrodes. j) Cycling curves as *E*_V_ and *E*_G_ of CNT–S and TiO_2_@VN–S cathodes. k) Cycling curve as *E*_V_ and *E*_G_ of TiO_2_@VN–S cathode in 10 and 7 µL mg^−1^_sulfur_ electrolyte at sulfur loading of 4.2 mg cm^−2^. l) Spider chart showing the performance comparisons between TiO_2_@VN–S and other reported cathodes constructed by carbon‐free heavy hosts.

The electrochemical performance of the TiO_2_@VN–S cathode was studied by pairing it with Li foil anode in the voltage range of 1.7–2.8 V (TiO_2_–S and VN–S cathodes were also tested for comparison). The typical galvanostatic discharge/charge curves of TiO_2_@VN–S, TiO_2_–S and VN–S at 0.2 C (1 C = 1675 mA g^−1^) are shown in **Figure** **4**a. The overpotential of TiO_2_@VN–S cathode is much smaller (141.4 mV) than those of VN–S (201.8 mV) and TiO_2_–S (208.3 mV) cathodes, suggesting the enhanced reaction kinetics accompanied with reduced polarization. Figure S18 (Supporting Information) exhibits the corresponding enlarged part of the galvanostatic charge curves of TiO_2_@VN–S, TiO_2_–S, and VN–S at 0.2 C. The onset potential of the TiO_2_@VN–S cathode (34.1 mV) is almost half of those for the TiO_2_–S (69.1 mV) and VN–S (57.4 mV) cathodes, indicating the improved redox reaction kinetics of the TiO_2_@VN–S cathode. The rate performances of the electrodes with the same amount of sulfur mass loading (1.6 mg cm^−2^) were measured. Note that the TiO_2_@VN–S displays the average discharge specific capacities of 1580, 1020, 882, 806, 686 and 594 mAh g^−1^ at 0.1 C, 0.2 C, 0.5 C, 1 C, 2 C, and 3 C, respectively, apparently superior to those of VN–S and TiO_2_–S cathodes, especially at high rate densities of 2 C and 3 C (Figure 4b). Besides, when the current density was returned to 2 C, 1 C, and 0.5 C after high‐rate cycling, extraordinarily reversible discharge capacities of 689, 797, and 868 mAh g^−1^ can be well attained for the TiO_2_@VN–S cathode, much better than those for TiO_2_–S and VN–S cathodes. Figure S19 (Supporting Information) displays the corresponding potential profiles of the three electrodes at various rates. Only the TiO_2_@VN–S cathode shows typical plateaus and relatively low polarization in all the discharge/charge curves, which are in good agreement with the CV results. The electrochemical impedance spectroscopy (EIS) measurements before cycling were further conducted. As presented in Figure 4c, all the Nyquist plots show one depressed semicircle at the high/medium‐frequency ranges and one sloping line at the low‐frequency range corresponding to the Warburg impedance.^[^ ^49^ ^]^ All cells have identical ohmic resistance (R_o_) values of about 2∼3 Ω, indicating that the cells were fabricated and measured under the same conditions. However, the TiO_2_@VN–S cathode presents the smallest charge transfer resistance (R_ct_) of 27 Ω, as compared to the VN–S cathode (30 Ω) and TiO_2_–S cathode (55 Ω), which manifests the accelerated charge transfer rate of TiO_2_@VN–S cathode due to its unique heterogeneous interface, thereby contributing to good rate performance.

In addition to the excellent rate capability, the TiO_2_@VN–S cathode also performs a remarkable stable cycling performance as well (all the cells were first activated for 3 cycles at 0.1C). As shown in Figure 4d, the TiO_2_@VN–S cathode possesses a high initial discharge capacity of 881 mAh g^−1^ at 0.5 C, and maintains an excellent reversible capacity of 795 mAh g^−1^ after 200 cycles, corresponding to a good capacity retention rate of 90.3%, which is much higher than the TiO_2_–S cathode (82.3%). Moreover, the long cycling performance at a higher current of 1C was also conducted (Figure S20, Supporting Information). Notably, a high initial capacity of 790 mAh g^−1^ together with a relatively low capacity decay rate of 0.054% per cycle for 500 cycles can be obtained by the TiO_2_@VN–S cathode. It is of vital importance for Li–S batteries to achieve high sulfur areal loading and low E/S ratio to satisfy the demand for high energy density. Thus, we further tested the cells with TiO_2_@VN–S cathodes under a considerable areal sulfur loading up to 4.2 and 7.6 mg cm^−2^ with a low E/S ratio of 10 and 8 µL mg^−1^_sulfur_, respectively, as shown in Figure 4e. Note that the TiO_2_@VN–S cathode exhibits a high initial areal capacity of 6.72 mAh cm^−2^ at the high sulfur loading of 7.6 mg cm^−2^, which can be maintained at 4.48 mAh cm^−2^ after 60 cycles with stable CE of around 98%. More strikingly, a 3 cm×3 cm flexible Li–S pouch cell was further assembled by pairing the TiO_2_@VN–S cathode (S loading: 14.4 mg) with a metallic Li anode, which can light up a visual “SCU” model including 60 reds light‐emitting diodes (LEDs) even under different bent states (Figure S21, Supporting Information).

As one of the fundamental demands for energy storage system such as electric automobiles and portable devices, the *E*_V_ of Li–S batteries is supposed to deserve more or at least as much attention as the *E*_G_ and cycle stability. However, most reported carbonaceous hosts show high surface area and low density, which are undesirable to satisfy high *E*_G_ and high *E*_V_ simultaneously. To this end, we further conducted the true density test for the engineered TiO_2_@VN heterostructure host, which shows its density as high as 5.01 g cm^−3^. Accordingly, the measured tap density of the corresponding sulfur‐loaded composite (i.e., TiO_2_@VN–S) can reach up to 1.52 g cm^−3^, as shown in Figure 4f, which is much higher than those of TiO_2_–S, VN–S, and pure S (1.2, 1.19, and 1.2 g cm^−3^ respectively). More notably, the tap density of TiO_2_@VN–S composite also significantly surpasses that of commercial carbon nanotubes‐loaded sulfur composite (CNT–S) (1.52 g cm^−3 ^vs0.67 g cm^−3^), as well as those of most conventional carbon/sulfur composites (usually <1.0 g cm^−3^). This makes it facile to prepare the densely compacted TiO_2_@VN–S electrode with a thickness of ≈20 µm, which is much smaller than that of the CNT–S cathode (≈34 µm) with the same mass loading (Figure 4g,h). Accordingly, the whole TiO_2_@VN–S cathode including the conductive agent and binder earns a high density (1.64 g cm^−3^), being about 1.7–folds higher than that for the CNT–S cathode (0.96 g cm^−3^), meanwhile the cathode porosity of the TiO_2_@VN–S is calculated as low as ≈30 vol%, being nearly half that of the CNT–S (≈54 vol%), as shown in Table S4 (Supporting Information). When further increasing the sulfur loading to 4.2 mg cm^−2^, the TiO_2_@VN–S cathode still keeps a relatively thinner thickness of ≈51 µm (Figure 4i), thus producing a desired higher cathode density of 1.69 g cm^−3^ alongside a lower cathode porosity of ≈28 vol% (Table S4, Supporting Information). As such, the corresponding *E*_V_ can be calculated according to the total volume of the whole cathode (the detailed calculation methods described in Supporting Information). Figure 4j compares the volumetric energy density between the TiO_2_@VN–S and CNT–S cathodes under the areal sulfur loading of 1.6 mg cm^−2^ at 0.1C. Amazingly, the peak *E*_V_ of TiO_2_@VN–S cathode reaches up to 2735 Wh L^−1^_cathode_, almost 2.3 times of that for the CNT–S cathode (1196 Wh L^−1^_cathode_). Moreover, with the elevated areal sulfur loading of 4.2 mg cm^−2^ and reduced E/S ratio of 10 µL mg^−1^_sulfur_ (the bottom half of Figure 4k), the TiO_2_@VN–S cathode can deliver high peak *E*_V_ (1696 Wh L^−1^_cathode_) and *E*_G_ (997 Wh kg^−1^_cathode_) based on the volume and mass of whole cathode at the areal current density of 0.5 mA cm^−2^ and keep a considerable *E*_V_ of 1275 Wh L^−1^_cathode_ at 1 mA cm^−2^ with ≈88% high capacity retention rate after 200 cycles (Figure 4k), which is comparable to most of the previously reported carbon‐based S cathodes (Table S5, Supporting Information). Even decreasing the amount of electrolyte further to 7 µL mg^−1^ (the up half of Figure 4k), a high volumetric energy density as high as 1633 Wh L^−1^_cathode_ can be still released at 0.2 mA cm^−2^, and also maintains around 1300 Wh L^−1^_cathode_ at an increased current density of 0.5 mA cm^−2^ accompanying with a high *E*_G_ of 767 Wh kg^−1^_cathode_ (Figure 4k). A spider chart was plotted in Figure 4l to expound the comprehensive performance features of our work compared with other recently reported composite cathodes with heavy hosts in terms of key parameters including tap density, *E*_V_, mass loading, E/S ratio and cycling performance.^[^ ^25^, ^29^, ^50^, ^51^, ^52^ ^]^ Hereinto, the TiO_2_@VN–S is more competitive than the majority of cathode materials, especially with respect to high tap density, superior *E*_V_, and long‐cycling stability. More notably, even after loading a higher sulfur content of 80.3 wt% in the TiO_2_@VN heterostructure host (denoted as TiO_2_@VN–S–80), the resultant TiO_2_@VN–S–80 cathode still shows a relatively thin thickness of around 23 µm with decent cathode density and porosity (1.4 g cm^−3^/32.8 vol%), thus giving rise to the desired initial *E*_V_ (1289 Wh L^−1^_cathode_), *E*_G_ (1085 Wh kg^−1^_cathode_), and superb capacity retention rate of 90.4% after 200 cycles at 0.5 C with the sulfur loading of 1.8 mg cm^−2^ (Figure S22, Supporting Information).

**Figure 5:**
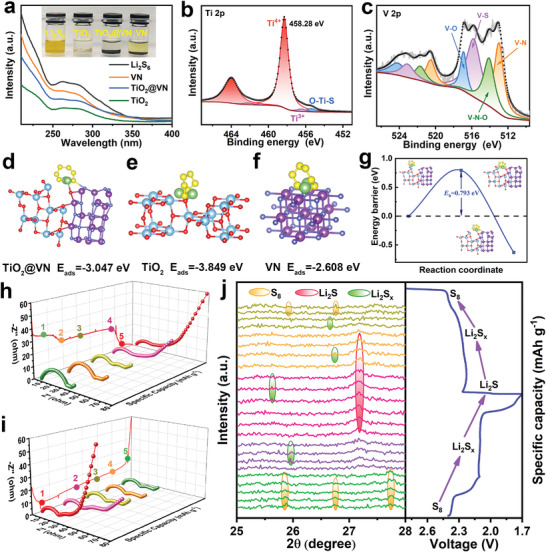
a) Visual observation of Li_2_S_6_ adsorption on TiO_2_, VN, and TiO_2_@VN: optical photos of Li_2_S_6_ adsorption test and UV–vis spectra of the supernatant. High‐resolution XPS spectra of b) Ti 2p and c) V 2p for TiO_2_@VN after interact with Li_2_S_6_. The optimized configurations of Li2S6 absorbed on d) TiO_2_@VN, e) TiO_2,_ and f) VN with corresponding binding energy. g) Diffusion barrier of Li_2_S_6_ on the TiO_2_@VN. h–i) Ex situ EIS plots and j) In situ XRD patterns of TiO_2_@VN–S at various charge and discharge states.

Effective immobilization of LiPS at the cathode side is crucial for remitting the shuttling issues in Li–S batteries. To identify the anchoring ability of TiO_2_@VN heterostructure towards LiPS, visualized Li_2_S_6_ adsorption experiments were performed by adding 10 mg VN, 20 mg TiO_2_@VN, and 40 mg TiO_2_ powder into the prepared Li_2_S_6_ solution, respectively, based on the approximately same specific surface area of three samples. As observed in the inset of **Figure** **5**a, the deep yellow Li_2_S_6_ solution in the presence of TiO_2_@VN becomes nearly transparent after 12 h, similar to a solution containing TiO_2_, indicating that this heterostructure inherits the strong adsorption capability from TiO_2_. Comparatively, the solution with the addition of VN still presents a slightly yellow color, suggesting the relatively weaker adsorption of VN than TiO_2_. This can be affirmed by the UV−vis adsorption spectra of the residual concentration of polysulfides in the above electrolyte solution after adsorption (Figure 5a). XPS analysis of TiO_2_@VN after adsorbing Li_2_S_6_ was conducted to further demonstrate the strong adsorption ability of the heterostructure. As seen from the S 2p spectrum (Figure S23, Supporting Information), six well‐distinguished pairs of peaks of S 2p at 163.1, 164.3, 166.9, 168.2, 162.1, and 160 eV can be attributed to the terminal (S_T_^−1^), bridging sulfur (S_B_^0^), thiosulfate, polythionate, Ti—S, and V—S respectively. Besides, Ti—S and V—S bonds are also observed in the XPS spectrums of Ti 2p and V 2p (Figure 5b,c). Note that the Ti 2p_3/2_ peak at 458.48 eV (Figure 2c) shifts to the position with lower binding energy at 458.28 eV (Figure 5b) after Li_2_S_6_ adsorption, which indicates the evident chemical interactions between TiO_2_@VN and LiPS.^[^ ^46^ ^]^ To gain deep insight into the adsorption mechanism, a DFT calculation was conducted. Figure 5d–f exhibits the optimized configurations of Li_2_S_6_ adsorption on the surface of TiO_2_@VN, TiO_2_, and VN. Among them, due to the strong Ti—S bonds, TiO_2_@VN and TiO_2_ exhibit much larger binding energy of –3.047 and –3.849 eV than that of single VN (–2.608 eV) with regard to the combination of Li_2_S_6_, which is consistent with the adsorption experiments. The diffusion pathway and barrier of Li_2_S_6_ on the TiO_2_@VN were also simulated, as shown in Figure 5g. The migration barrier over TiO_2_@VN is 0.793 eV, much lower than that on the TiO_2_ (101) surface (1.20 eV),^[^ ^18^ ^]^ implying that more smooth and rapid diffusion of Li_2_S_6_ can be realized at the interface of TiO_2_@VN heterostructure. Accordingly, by incorporating the VN with high electrical conductivity into the strong adsorptive TiO_2_ plus the presence of interfacial built‐in electric field, the heterostructured TiO_2_@VN ensure favorable electron transfer, effective spatial dispersion and immobilization of LiPS as well as bidirectional consecutive conversion during the charge‐discharge process.

The ex situ EIS measurements were conducted to monitor the electron/ion transfer of the TiO_2_@VN–S cathode during the whole discharge/charge process. As seen in Figure 5h, there clearly exists another small semicircle in the middle frequency region from 1 to 3 steps of the discharge process, which is corresponding to the interface contact resistance between soluble LiPS and electrode. Moreover, the sluggish Li‐ion transport in the steps of 1–3 is related to the increased viscosity of the electrolyte due to the soluble LiPS in the electrolyte. However, the LiPS in the electrolyte are transformed into the insoluble Li_2_S_2_/Li_2_S and thus the viscosity of the electrolyte decreases, resulting in the enhanced Li‐ion transport in steps 4–5.^[^ ^53^ ^]^ Expectedly, the solid electrolyte interface (SEI) resistance decreases when the discharge process is near the end (step 5), implying that the insulated Li_2_S_2_/Li_2_S is gradually formed and can be effectively accommodated with the TiO_2_@VN hosts. The change of the resistance during the charge process is given in Figure 5i, which is the opposite to that of the discharge process. To real‐time monitor the solid/liquid phase transition of the TiO_2_@VN–S cathode in the discharge/charge process, in situ XRD measurements were carried out (Figure 5j). Initially, the characteristic peaks of orthorhombic *α*–S_8_ (JCPDS No. 08‐0247) can be easily detected. As the discharge takes place, the intensities of S_8_ peaks gradually fade away accompanied by the signature of long‐chain LiPS formed at 25°–26°. In the meantime, a new characteristic peak of crystalline cubic Li_2_S (JCPDS No. 23‐0369) appears at the start of the lower discharge plateau, manifesting that the liquid‐solid phase transformation emerges at the initial stage of discharge. Additionally, the intensities of Li_2_S peak increase continuously and finally reach the maximum values along with the weakened signals of LiPS, demonstrating the complete transformation of long‐chain LiPS to Li_2_S. In the subsequent charging process, the highly reversible oxidation processes of sulfur species, i.e., Li_2_S → LiPS → *β*–S_8_, can be apparently observed.

In order to better illustrate the superiority of TiO_2_@VN heterostructure, a schematic illustration of the role of TiO_2_, VN and TiO_2_@VN heterostructure in tuning S chemistry is shown in Figure S24 (Supporting information). As clearly seen, the single‐component TiO_2_ is able to exhibit strong chemical adsorption properties toward LPS species. However, due to the intrinsically low electrical conductivity of TiO_2_, it is hard for a mass of the immobilized LiPS to effectively contact with electrons, thus making them cannot being fully involved in the subsequent electrochemical reactions, and finally resulting in undesirable aggregation and remaining of LiPS on the TiO_2_ surface with low conversion efficiency. Comparatively, the single‐component VN possesses a higher electrical conductivity, which can provide fast electron‐transfer pathways to the adsorption sites of LiPS. However, its adsorption ability toward LiPS is much weaker than that of TiO_2_, leading to an obvious dissolution of LiPS and severe shuttling, so as to compromise the good catalysis effect of VN. By contrast, the designed TiO_2_@VN heterostructure integrates strong adsorptive TiO_2_ with high conductive VN, and concurrently yields a built‐in electric field which could favor a spatially optimized distribution of active LPS species and enlarge the electroactive locations for catalytic conversion of polysulfides at the TiO_2_/VN interfaces.

**Figure 6:**
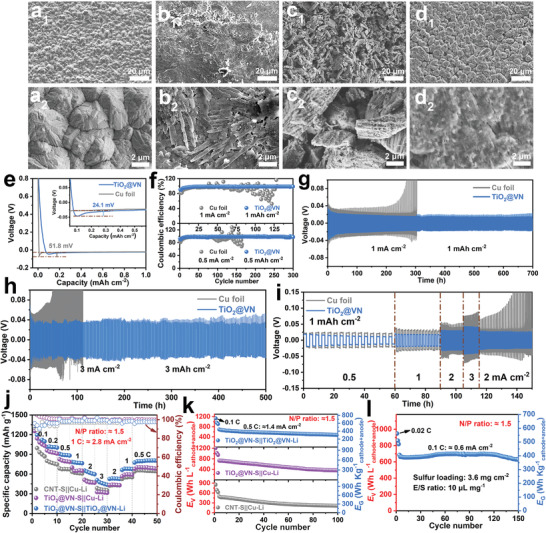
FESEM images of Cu foil and TiO_2_@VN electrodes a_1_,a_2_,c_1_,c_2_) before and b_1_,b_2_,d_1_,d_2_) after Li deposition after 100 cycles at a density of 1 mA cm^−2^ for 1 h. e) Voltage profiles of Li deposition on TiO_2_@VN and Cu foil electrodes at 0.5 mA cm^−2^. f) Coulombic efficiencies of Cu foil and TiO_2_@VN electrodes at various current densities with different cycling capacities. Voltage profiles of metal Li plating/stripping in Li|Cu–Li and Li|TiO_2_@VN–Li symmetric cells with an areal capacity of g) 1 mAh cm^−2^ and h) 3 mAh cm^−2^. i) Rate performance comparison at various current densities from 0.5 to 3 mA cm^−2^. Comparison of the j) rate and k) cycling performance of CNT–S||Cu–Li, TiO_2_@VN–S||Cu–Li and TiO_2_@VN–S||TiO_2_@VN–Li full cells at sulfur loading of 1.6 mg cm^−2^. l) Cycling performance of TiO_2_@VN–S||TiO_2_@VN–Li full cell at sulfur loading of 3.6 mg cm^−2^.

Besides concerning the S cathodes, the unwelcome Li dendrite issues commonly caused by nonuniform Li^+^ flux at the Li metal anodes also cast a critical challenge for practical working Li–S full batteries. However, the affection of heterostructures on the Li‐metal anode has rarely been investigated to date. In this regard, we further explored the effectiveness of the TiO_2_@VN as the anode host in tuning the lithium stripping/plating and the suppression of Li‐dendrite propagation. The surface morphologies of Cu foil modified by TiO_2_@VN or not were first compared with a Li‐plating capacity of 3 mAh cm^−2^ after Li‐stripping/plating for 100 cycles under a plating capacity of 1 mAh cm^−2^. The messy morphology with a thorn‐like structure on the surface of the bare Cu foil electrode manifests the obvious and undesirable dendrite growth in the Cu–Li electrode (**Figure** **6**a_1_,a_2_,b_1_,b_2_). In contrast, it presents a dendrite‐free morphology with a relatively smooth and dense surface for the TiO_2_@VN modified electrode, due to the Li dendrite growth along the direction as the TiO_2_@VN fibers, indicating the lithiophilic nature of TiO_2_@VN heterostructure, as displayed in Figure 6c_1_,c_2_,d_1_,d_2_. Uniform Li deposition is in favor of forming a steady SEI layer inhabiting the negative interaction between the electrode and the electrolyte.^[^ ^54^, ^55^, ^56^ ^]^ To demonstrate the function of TiO_2_@VN as anode hosts on the behavior of Li deposition, nucleation overpotentials (µ_ŋ_) on the bare Cu foil and TiO_2_@VN electrodes were employed. Correspondingly, the voltage‐capacity curves at 0.5 mA cm^−2^ are shown in Figure 6e. When Li was deposited on the TiO_2_@VN electrode, it presents a smaller nucleation overpotential of 24.1 mV than that of Cu foil (51.8 mV), suggesting the supportive Li nucleation associated with the lithiophilic property of TiO_2_@VN hosts. In addition, the coulombic efficiency (CE) was further measured to investigate the reversibility of the Li‐stripping/plating process. The CE values of Cu foil and TiO_2_@VN electrodes at different current densities are compared in Figure 6f. Obviously, the Cu foil electrode exhibits a stable CE only for a few cycles and then suffers from a dramatic fluctuation due to the uncontrollable Li deposition. By contrast, the TiO_2_@VN electrode keeps a superb and steady CE of 99.98% for 300 cycles at 0.5 mAh cm^−2^ (150 cycles at 1 mAh cm^−2^), indicating the enhanced reversibility of the Li‐stripping/plating process on the TiO_2_@VN electrode.

Symmetric cells were further fabricated to evaluate the long‐term cycling stability of the Li metal anode. A Li areal capacity of 3 mAh cm^−2^ was first pre‐plated on the Cu foil and TiO_2_@VN electrodes before cycling. The Li|TiO_2_@VN–Li symmetric cells with an areal capacity of 0.5 mAh cm^−2^ at 0.5 mA cm^−2^ display superior cycling stability over 900 h with an overpotential lower than 20 mV, as shown in Figure S25 (Supporting Information). Even at higher current densities (1 mA cm^−2^, 3 mA cm^−2^) and capacities (1 mAh cm^−2^, 3 mAh cm^−2^), the TiO_2_@VN anode still exhibits low voltage hysteresis of 24 mV over 700 h (Figure 6g) and 35 mV over 500 h (Figure 6h), respectively, without obvious voltage fluctuation. However, the bare Cu foil electrode shows obvious voltage fluctuation along with larger voltage hysteresis, which is attributed to the unstable lithium/electrolyte interface and the growth of lithium dendrite. Furthermore, the electrochemical performances of the Li|TiO_2_@VN–Li and Li|Cu–Li symmetric cells at a series of current densities with a fixed areal capacity of 1 mAh cm^−2^ were also carried out. As depicted in Figure 6i, the Li|TiO_2_@VN–Li symmetric cell delivers low voltage hysteresis and remarkable rate performance, much better than the Li|Cu–Li cell. The outstanding performance demonstrates the homogeneous deposition of Li with stable SEI structure when the TiO_2_@VN heterostructure as the Li anode hosts.

Encouraged by the impressive dual‐function of the TiO_2_@VN heterostructure as both sulfur and Li metal hosts, Li–S full cells were consequently assembled by pairing the TiO_2_@VN–S cathode with the TiO_2_@VN–Li anode, where the lithium excess is controlled around 50% (i.e., N/P≈1.5) while the thickness of TiO_2_@VN–Li with an areal capacity of 4 mAh cm^−2^ is measured as ≈23 µm (Figure S26a, Supporting Information). As shown in Figure 6j, the TiO_2_@VN–S||TiO_2_@VN–Li full cell exhibits superior rate capabilities of 1285, 1010, 904, 782, 652, 506 mAh g^−1^ at 0.1 C, 0.2 C, 0.5 C, 1 C, 2 C, and 3 C (1C: ≈2.8 mA cm^−2^), respectively, significantly outperforming the counterpart full cells (TiO_2_@VN–S||Cu–Li, CNT–S||Cu–Li). When recovering the current density to 0.5 C, a high discharge capacity of 792 mAh g^−1^ can be still retained for the TiO_2_@VN–S||TiO_2_@VN–Li full cell. Figure 6k shows the cycling performance of the three full cells. After being activated at 0.1 C for three cycles, the TiO_2_@VN–S||TiO_2_@VN–Li full cell is able to deliver a considerable electrode‐level *E*_V_ of approaching 700 Wh L^−1^_cathode+anode_ (based on total volumes of cathode and anode) at 0.5 C (≈1.4 mA cm^−2^). Especially as compared to the conventional CNT–S||Cu–Li full cell, both the TiO_2_@VN–S||TiO_2_@VN–Li and TiO_2_@VN–S||Cu–Li full cell show much higher *E*_V_ and *E*_G_, indicating the superiority of carbon‐free fibrous TiO_2_@VN heterostructure in behaving the dual‐capable S and Li hosts. Notably, because the thickness of the Cu–Li anode is smaller than that of the TiO_2_@VN–Li anode (Figure S26b, Supporting Information), the *E*_V_ of the TiO_2_@VN–S||Cu–Li full cell is higher than that of the TiO_2_@VN–S||TiO_2_@VN–Li full cell in the first 35 cycles, but it drops dramatically in the following cycles. Contrarily, the TiO_2_@VN–S||TiO_2_@VN–Li full cell can exhibit a decent capacity retention of 71% after 100 cycles, which is much higher than the TiO_2_@VN–S||Cu–Li and CNT–S||Cu–Li full cells with only 49% and 41% capacity retention, respectively, as illustrated in Figure 6k. The above comparison results demonstrate that the stable operation of Li–S full cell heavily relies on the rational electrode design upon the S cathode and the Li‐metal anode, together with concurrently regulated Li and S electrochemistry properties.

More practically, we further assembled and tested the feasibility of TiO_2_@VN–S||TiO_2_@VN–Li full cell with a higher sulfur loading of 3.6 mg cm^−2^ at a low E/S ratio of 10 µL mg^−1^_sulfur_ (N/P ratio still kept around 1.5)_._ Note that the thicknesses of TiO_2_@VN–S cathode and TiO_2_@VN–Li anode in this high‐loading full cell are measured to be around 44 µm and 28 µm, respectively (Figure S27, Supporting Information), which consequently enable the TiO_2_@VN–S||TiO_2_@VN–Li full cell to achieve high initial electrode‐level *E*_V_/*E*_G_ of 951 Wh L^−1^_cathode+anode_ /562 Wh kg^−1^_cathode+anode_ based on the cathode and the anode at a low current density of 0.02 C (≈0.12 mA cm^−2^), while even at an elevated current density of 0.1 C, it still can attain decent energy densities of 687 Wh L^−1^_cathode+anode_/407 Wh kg^−1^_cathode+anode_ as well as a satisfying cycling performance over 150 cycles (Figure 6l). Our work manifests that the elaborately designed and constructed S/Li host configuration is of fundamental importance toward the pursuit of practical high energy density Li–S full batteries.

## 3 Conclusion

In summary, we have designed and engineered a carbon‐free dual‐capable fibrous host configuration made up of high‐density, compact TiO_2_@VN heterostructure simultaneously applied for both S cathode and Li anodes toward Li–S batteries. As a kind of heavy heterostructure host for S loading, the TiO_2_@VN is proved to work on fabricating high‐tap‐density TiO_2_@VN–S composite as well as highly dense cathodes. Moreover, such interfacial engineered heterostructure via a smart selective nitridation strategy is demonstrated experimentally and theoretically to establish a built‐in electric field at the TiO_2_/VN interfaces, thus ensuring the LiPS immobilization, dispersion and utilization along with rapid redox kinetics. The synergistic merits from TiO_2_@VN heterostructure bestow the S electrodes with enhanced electrochemical performance in terms of high rate capability, good cycling stability, decent areal capacity, and outstanding volumetric energy density which are competitive with state‐of‐the‐art properties in Li–S batteries. When used for hosting the Li metal, the heterostructured TiO_2_@VN with a specific lithiophilic feature is beneficial to support and tune the Li nucleation with restrained lithium dendrite growth. Combining the impressive dual‐function of the heterostructure for both S and Li electrodes, the Li–S full cell paired the compact TiO_2_@VN–S cathode with dense TiO_2_@VN–Li anode exhibits enhanced energy density, particularly under the operation with extremely low lithium excess in the anode. We hope this work can provide a viable strategy for exploring dual‐capable heavy hosts to realize high energy density Li–S batteries for future applications.

## Conflict of Interest

The authors declare no conflict of interest.

## Supporting information

Supporting Information

## Data Availability

The data that support the findings of this study are available in the supplementary material of this article.
