# Supplementary material for: Selective Nitridation Crafted a High‐Density, Carbon‐Free Heterostructure Host with Built‐In Electric Field for Enhanced Energy Density Li–S Batteries
Source: Adv Sci (Weinh). 2022 Jun 16;9(23):2201823. doi: 10.1002/advs.202201823 (PMC9376747; doi:10.1002/advs.202201823)
Supplement: Supplementary file 1 — Supporting Information [file ADVS-9-2201823-s001.pdf]

© 2022 Wiley-VCH GmbH

Supporting Information

Selective Nitridation Crafted a High-Density, Carbon-Free Heterostructure Host with Built-In Electric Field for Enhanced Energy Density Li–S Batteries

Hongmei Wang, Yunhong Wei, Guochuan Wang, Yiran Pu, Li Yuan, Can Liu, Qian Wang, Yun Zhang and Hao Wu**

H. Wang, G. Wang, Y. Pu, L. Yuan, Prof. C. Liu, Q. Wang, Prof. Y. Zhang, Prof. H. Wu
Engineering Research Center of Alternative Energy Materials & Devices, Ministry of Education, College of Materials Science and Engineering
Sichuan University
Chengdu, Sichuan, 610065, China
E-mail: liucan@scu.edu.cn; hao.wu@scu.edu.cn
Dr. Y. Wei
Hefei National Laboratory for Physical Sciences at the Microscale, Department of Applied Chemistry
University of Science and Technology of China
Hefei, 230026, China
E-mail: weiyunhong@ustc.edu.cn

Section SI. Experimental Section

Chemicals and materials

Collagen fibers (CFs) were prepared according to the previously reported method.[S1-S3] Chemically pure $NaHCO_3$, $Ti(SO_4)_2$ and NH_4VO_3 were purchased from the Jinshan Chemical Reagent Corporation (Chengdu, China). Analytical grade NaCl and H_2SO_4 were obtained from the Chengdu Kelong Chemical Reagent Factory. Sulfur (99.99 wt%) and Li_2S were from Aladdin (Shanghai, China). 1-methyl-2-pyrrolidinone (NMP, 99.5 wt%), bis-(triuoromethane) sulfonimide lithium salt (LiTFSI, 99.95 wt%), $LiNO_3$ (99.99 wt%), 1, 2-dimethoxyethane (DME, 99.5 wt%), 1, 3-dioxolane (DOL, 99.8 wt%) were purchased from Sigma-Aldrich (Darmstadt, Germany).

Synthesis of the Ti^{4+}/VO_3^-@CFs, Ti^{4+}@CFs, and VO_3^-@CFs intermediates

First, 15.0 g collagen fibers (CFs) and 6.0 g NaCl were uniformly dispersed into 300 ml deionized water and stirred for 6 h at room temperature. After that, the pH value of the dispersion was first adjusted to 1.8 by using 1.0 M H_2SO_4 in order to prevent from unhopeful hydrolysis of subsequently added 15 g $Ti(SO_4)_2$. Then Ti^{4+}-immobilized CFs (Ti^{4+}@CFs) were obtained by slowly increasing the pH value to 4.0 using saturated $NaHCO_3$ solution and following vigorous stirring for 12 h at 40 °C. Afterwards, the prepared Ti^{4+}@CFs and 5 g NH_4VO_3 were added into a round bottomed flask with 300 mL H_2O stirring for 1 h. Subsequently, the pH value was decreased to 4.0 by 0.3 M H_2SO_4 and stirring for 12 h at 40 °C. After washing and dehydrating by freeze drying, the Ti^{4+}/VO_3^--co-immobilized CFs (Ti^{4+}/VO_3^-@CFs) were obtained. For comparison, the Ti^{4+}-immobilized and VO_3^--immobilized CFs, denoted as Ti^{4+}@CFs and VO_3^-@CFs, respectively, were also prepared by the similar method only by using single metallic salt Ti^{4+} for Ti^{4+}@CFs and VO_3^- for VO_3^-@CFs.

Synthesis of the TiO_2@VN, TiO_2, and VN host materials

First, the as-prepared Ti^{4+}/VO_3^--CFs precursors were activated at 300 °C for 2 h and heated to 500 °C with a heating rate of 3 °C min^{-1} for 4 h in air atmosphere. Then, following by the annealing at 600 °C for 2 h in NH_3 atmosphere, the resultant TiO_2@VN were obtained. For comparison, the two control samples, TiO_2 and VN, were also prepared separately by the same process as the preparation of TiO_2@VN.

Synthesis of the TiO_2@VN–S, TiO_2–S, and VN–S composites

To prepare TiO_2@VN–S composites, the as-prepared TiO_2@VN and sulfur were firstly mixed uniformly by the ratio of 3:7. Then, the mixed powder was transferred into the tubular furnace and heated at 155 °C for 12 h under Ar atmosphere. Before cooling down, the as-prepared sample was annealed at 250 °C for 2 h to remove the redundant sulfur on the external surface of the TiO_2@VN. Finally, the TiO_2@VN–S composites were obtained. For comparison, the TiO_2–S and VN–S composites were obtained in the same way.

Electrochemical measurements of S-composite cathode

The TiO_2@VN–S cathode was fabricated by mixing 70% TiO_2@VN–S composites with 20% Super P and 10% PVDF in NMP to form homogeneous slurry and then coated on the Al/C foil dried at 60 °C for 12 h, shaped into circular plates with 12 mm. Then, the cathode was assembled with Li foil anode and Celgard 2400 separator into CR2032 coin cells inside glove box with H_2O and O_2 below 0.1 ppm. For the pouch cell assembly, the TiO_2@VN–S cathode and lithium anode were cut into 3×3 cm pieces. The sulfur loading of the cathode in the pouch cell was about 1.6 mg cm^{-2}. Electrolyte was using 1 wt % $LiNO_3$ additive and 1.0 M LiTFSI in a mixture of DOL and DME with volume ratio of 1:1. The galvanostatic charge/discharge tests were performed with a multi-channel battery test system (Neware CT–3008W, China) in voltage window of 1.7~2.8 V. Cyclic voltammetry (CV) measurement was conducted using a PARSTAT multichannel electrochemical workstation (Princeton Applied Research, USA). Electrochemical impedance spectrums (EIS) were performed with a frequency range from 100

kHz to 0.1 Hz and AC amplitude of 5 mV. For the in-situ XRD measurement, the cathode was prepared by coating the slurry onto Al foil and the in-situ XRD cell was cycled at 1.7~2.8 V at 0.05 C rate with a Landt CT2001A battery test system.

Li metal anode tests

TiO_2@VN–Li working electrode was prepared by mixing 90 wt% TiO_2@VN and 10 wt% PVDF in NMP to form the homogeneous slurry and then coated on Cu foil, which was punched into a disk with a diameter of 12 mm. The lithium foil was used as the counter electrode in CR2032 coin cell and 40 μL above-mentioned electrolyte was added in each cell. Assembled cells were primarily cycled from 0 to 1 V for 5 cycles at a current density of 50 μA to stabilize the interface.

Li–S full cell tests

Li–S full cells were assembled by using TiO_2@VN–S as the cathode, TiO_2@VN–Li as the anode, and the Celgard 2400 separator into a CR2032 coin cell. The sulfur loading was controlled at 1.6 mg cm^{-2}, while the TiO_2@VN–Li anode was prepared by pre-plating 4 mAh cm^{-2} of Li onto the TiO_2@VN-coated Cu foil. For a higher sulfur loading of 3.6 mg cm^{-2}, the TiO_2@VN–Li anode was prepared by pre-plating 9 mAh cm^{-2} of Li onto TiO_2@VN-coated Cu foil. The E/S ratio was 15 μL $mg^{-1}{}_{S}$ and 10 μL $mg^{-1}{}_{S}$ at the sulfur loading of 1.6 and 3.6 mg cm^{-2} respectively. The contrast sample was assembled using CNT–S cathode and Cu–Li anode without TiO_2@VN modification. The galvanostatic charge/discharge test was performed with Neware CT–3008W tester at different current densities within a voltage window of 1.7~2.8 V.

Lithium polysulfide adsorption tests

The 5.0 mmol L^{-1} Li_2S_6 solution was prepared by dissolving stoichiometric amounts of S and Li_2S with a molar ratio of 5:1 in a mixed solvent of 1, 2-dioxolane (DOL) and dimethoxymethane (DME) (1:1 by volume) in an argon-filled glove-box. Typically,

visualized adsorption tests were carried out by adding 20 mg of TiO_2@VN, 40 mg of TiO_2 and 10 mg of VN composites (based on approximately the same surface area) into 3 mL of the as-prepared Li_2S_6 solution respectively. The concentration of the residual lithium polysulfide in the solution was determined by using a UV-vis absorption spectrophotometry (UV-vis, Shimadzu UV 3600).

Symmetric cell tests

TiO_2@VN and polyvinylidene fluoride (PVDF) were mixed by a weight ratio (9:1) in N-methyl-pyrrolidone (NMP) solvent, and then coated on the Al/C foil. After drying at 60 °C for 12 h, the electrode was punched into disks with a diameter of 12 mm. The active material loading was controlled at about 1.5 mg cm^{-2}. Li_2S_6 symmetric cells were assembled by employing two identical TiO_2@VN electrodes, Celgard 2400 separator, and 40 μL of Li_2S_6 electrolyte (0.125 M). The CV tests were performed between −1.0 and 1.0 V by a PARSTAT electrochemical workstation (Princeton Applied Research, USA).

Nucleation and dissolution behavior of Li_2S studies

To investigate the liquid-solid reaction kinetics, the TiO_2@VN coated on Al/C foil was assembled into the CR2032 coin cell by paring Li foil as anode. 20 μL Li_2S_8 catholyte (composed of 0.3 M Li_2S_8 and 1.0 M LiFSI dissolved in tetraglyme solution) was added into the cathode side and 20 μL tetraglyme solution was added into the anode side. For the nucleation of Li_2S studies, the cell was galvanostatically discharged to 2.06 V, and then discharged potentiostatically at 2.05 V until the current was lower than 10^{-5} A. To analyze the Li_2S dissolution behavior, fresh cells were first discharged at a current of 0.10 mA to 1.80 V, and subsequently discharged at 0.01 mA to 1.80 V for full transformation of S species into solid Li_2S. After this discharge, cells were potentiostatically charged at 2.40 V for the dissolution of Li_2S into LiPS until the charge current was below 10^{-2} mA.

Materials characterizations

The structure and phase composition analyze of all materials were tested by X-ray diffraction (XRD) patterns conducted with a Bruker DX–1000 diffractometer with Cu Ka radiation (λ=1.54178 Å, Philips X'pert TROMPD). The density of TiO_2@VN composites was tested by fully automatic true density analyzer (American Mike automatic true density tester AccuPyc II 1340). Electronic conductivity tests of TiO_2 @VN, TiO_2 and VN composites were measured by four-probe direct current method using ST2253 type digital four-probe tester. The field-emission scanning electron microscopy (FESEM, HitachiS-4800) and transmission electron microscopy (TEM, JEOL JEM–2100F) coupled with energy-dispersive X-ray (EDX, Oxford Instrument) spectroscopy were used to characterize the morphology and microstructure. To determine the chemical element composition, X-ray photoelectron spectroscopy (XPS) tests were measured by AXIS Ultra DLD, Kratos. American PE Avio 200 inductively coupled plasma optical emission spectrometer (ICP–OES) was used to analyze the metal contents in the samples. The ICP–OES results of three TiO_2@VN samples are displayed in **Table S1**. Ultraviolet Photoelectron Spectroscopy (UPS) was collected on Thermo Scientific ESCALAB Xi+ equipped with ultraviolet photoelectron spectroscopy (Hel (21.2 eV). The work function (E_Φ) is calculated by the following formula:[S4] $E_\Phi = h\nu - (E_{cutoff} - E_f)$, $h\nu = 21.2$ eV, $E_f = 0$. Nitrogen adsorption/desorption measurements were performed on a Kubo–X1000 analyzer (Beijing Builder Electronic Technology Co., Ltd). Thermogravimetric analysis (TGA, METTLER TOLEDO, USA) was carried out at a temperature range of 25–600 °C with a heating rate of 10 °C min^{-1} in nitrogen atmosphere to analyze the content of sulfur in the composites.

Table S1 ICP–OES results of three TiO_2@VN samples

Samples	Ti 334.94 nm (mg g^{-1})	V 292.46 nm (mg g^{-1})	wt% (TiO_2:VN)
#1	4.03×10^2	2.53×10^2	67.5:32.5
#2	3.93×10^2	2.49×10^2	67.4:32.6
#3	3.83×10^2	2.33×10^2	66.8:33.2

Density functional theory calculation

The structural optimizations and electronic structure calculations are performed based on density functional theory (DFT) as implemented in the Vienna Ab Initio Simulation Package (VASP) code,[S5] based on the projector augmented wave (PAW) method with a cutoff energy of 600 eV.[S6] All of configurations of TiO_2@VN based materials were fully optimized.[S5] The generalized gradient form (GGA) of the exchange-correlation functional (Perdew-Burke-Ernzerhof 96, PBE) was adopted.[S7, S8] A revised Perdew-Burke-Ernzerhof generalized gradient approximation (PBEsol)[S9, S10] was used for the exchange-correlation. PBEsol functional has been introduced to improve the equilibrium properties of solids.[S11] Valence-core interactions were described by projector-augmented-wave (PAW) pseudopotentials.[S12] The Brillouin zone sampling is carried out using the (3×3×1) Monkhorst-Pack grids for surface and Gamma for the structure.[S6] The convergence tolerance of energy is 1×10^{-5} eV, maximum force is 0.002 eV $Å^{-1}$, and maximum displacement is 0.002 Å.[S6]

Lithium-ion diffusion coefficients (D_{Li^+})

The D_{Li^+} can be calculated by the classic Randles–Sevcik equation:

$$I_p = (2.69 \times 10^5) n^{1.5} A D_{Li^+}{}^{0.5} C v^{0.5}$$

where *Ip* is the peak current (mA), *n* is the charge transfer number per reaction species (n=2), A is the geometric electron area (1.13 cm^2 here), C is the concentration of lithium ions

(10^{-3} mol cm^3 here), and v is the scan rate (V s^{-1}). According to the above equation, the calculated slope values and D_{Li^+} are listed in **Table S2**.

Table S2 Lithium-ion diffusion coefficients (D_{Li^+}) derived from CV curves.

**Samples**	**D_{Li^+} (cm^2 s^{-1})**		
	**Peak 1**	**Peak 2**	**Peak 4**
**TiO_2@VN-S**	6.01×10^{-8}	5.08×10^{-8}	4.34×10^{-7}
**VN-S**	4.62×10^{-8}	2.18×10^{-8}	4.10×10^{-7}
**TiO_2-S**	4.52×10^{-8}	2.01×10^{-8}	2.69×10^{-7}

Electrode porosity

The electrode porosity (ε) is determined from the relation:[S13]

$$\varepsilon = 1 - \frac{m_{areal}\left(\frac{w_{Sulfur} + w_{host} + w_{conductive} + w_{PVDF}}{\rho_{Sulfur} + \rho_{host} + \rho_{conductive} + \rho_{PVDF}}\right)}{L}$$

where m_{areal} (g cm^{-2}) is the mass loading of the electrode except current collector, L(cm) is the electrode thickness, and w (%) and ρ (g cm^{-3}) are the mass fraction and real density of every component, respectively.

Table S3 True density and mass fraction of every component.

**Characteristics**	**Sulfur**	**Host**		**Conductive agent (Super P)**	**PVDF**
		TiO_2@VN	CNT		
True density (g cm^{-3})	2.07	5.01	2.2	2.2	1.76
Mass fraction (wt%)	0.4865	0.2135	0.2135	0.2	0.1

Table S4 Porosity of TiO_2@VN–S and CNT–S electrodes.

**Characteristics**	**TiO_2@VN–S electrode**		**CNT–S electrode**	**TiO_2@VN–S–80 electrode**
Sulfur loading (mg cm^{-2})	1.6	4.2	1.6	1.8
Porosity (%)	30.1	28.3	53.5	32.8

Calculation of energy density

The cathode-level (based on the cathode) volumetric energy density (E_V) and gravimetric energy density (E_G) of half-cell was calculated according to the following equation:

$$E_v = \frac{m_{cathode} \times Q_{cathode} \times U}{d_{cathode} \times S}$$

$$E_G = Q_{cathode} \times U$$

where m is the mass of cathode (mg), Q is the capacity of the cathode (mAh g^{-1}), U is the voltage at half specific capacity during discharge (V), d is the thickness of cathode (μm), and S is the area of electrode (cm^{-122}).

The electrode-level (based on the cathode and the anode) volumetric energy density (E_V) and gravimetric energy density (E_G) of full-cell was calculated according to the following equation:

$$E_v = \frac{m_{cathode} \times Q_{cathode} \times U}{d_{cathode+cathode} \times S}$$

$$E_G = \frac{m_{cathode} \times Q_{cathode} \times U}{m_{cathode+anode}}$$

where m is the mass of cathode and anode (mg), Q is the capacity of the cathode (mAh g^{-1}), U is the voltage at half specific capacity during discharge (V), d is the total thickness of cathode and anode (μm), and S is the area of electrode (cm^{-2}).

In our experiment, the S fraction in the TiO_2@VN–S composites is 69.5%. The slurry spices for TiO_2@VN–S, Super P, PVDF is 7: 2: 1. The final ratio for S, TiO_2@VN, Super P, PVDF is 0.4865: 0.2135: 0.2: 0.1.

For the TiO_2@VN–S cathode in half cells with the sulfur loading of 1.6 mg cm^{-2} at a current density of 0.1 C, the E_V and E_G (based on the cathode) were calculated as follows:

$$E_v = \frac{m_{cathode} \times Q_{cathode} \times U}{d_{cathode} \times S}$$

$$= \frac{3.72\ mg \times 1626.4\ mAh\ g^{-1} \times 0.4865 \times 2.1\ V}{20\ \mu m\ \times 1.13\ cm^{-2}}$$

$$= 2735\ Wh\ L_{cathode}^{-1}$$

$$E_G = Q_{cathode} \times U$$

$$= 1626.4\ mAh\ g^{-1} \times 0.4865 \times 2.1\ V$$

$$= 1662\ Wh\ Kg_{cathode}^{-1}$$

For the TiO_2@VN–S cathode with the sulfur loading of 4.2 mg cm^{-2} at a current density of 0.5 mA cm^{-2}, the E_V and E_G (based on the cathode) were calculated as follows:

$$E_v = \frac{m_{cathode} \times Q_{cathode} \times U}{d_{cathode} \times S}$$

$$= \frac{9.8\ mg \times 976\ mAh\ g^{-1} \times 0.4865 \times 2.1\ V}{51\ \mu m\ \times 1.13\ cm^{-2}}$$

$$= 1696\ Wh\ L_{cathode}^{-1}$$

$$E_G = Q_{cathode} \times U$$

$$= 976\ mAh\ g^{-1} \times 0.4865 \times 2.1\ V$$

$$= 997\ Wh\ Kg_{cathode}^{-1}$$

For the TiO_2@VN–S||Cu–Li full cell with the sulfur loading of 3.6 mg cm^{-2} at a current density of 0.1 C, the electrode-level (based on the cathode + anode) volumetric energy density E_V and E_G were calculated as follows:

$$E_v = \frac{m_{cathode} \times Q_{cathode} \times U}{d_{cathode+anode} \times S}$$

$$= \frac{8.36\ mg \times 654\ mAh\ g^{-1} \times 0.4865 \times 2.1\ V}{(44 + 28)\ \mu m\ \times 1.13\ cm^{-2}}$$

$$= 687\ Wh\ L_{cathode+anode}^{-1}$$

$$E_G = \frac{m_{cathode} \times Q_{cathode} \times U}{m_{cathode+anode}}$$

$$= \frac{8.36\ mg\ \times 654\ mAh\ g^{-1} \times 0.4865 \times 2.1\ V}{8.36\ mg + 2.63\ mg + 2.75\ mg}$$

$$= 407\ Wh\ Kg_{cathode+anode}^{-1}$$

Where the mass of cathode is 8.36 mg, and the mass of anode is 5.38 mg (2.63 mg Li and 2.75 mg TiO_2@VN).

Table S5 Comparison of our work with other previously reported sulfur cathode materials.

Sulfur host	Sulfur content (wt%)	Current density	Sulfur loading (mg cm^{-2})	E_V (cathode–level, Wh L^{-1})	E/S ratio (μL mg^{-1})	Ref.
VO_2 HSs	71	0.1 C	0.8	2276	31.8	**[S14]**
TCD–TCS	67.6	0.05 C	4	3070	10	**[S15]**
3D carbon network	84	0.1 C	10.9	1833	6	**[S16]**
Active Mo_6S_8	45	0.05 C	6.2	1232	2.4	**[S17]**
$NiFeO_4$	82.5	0.1 C	1.0~1.3	1859	20	**[S18]**
$NiCoO_4$	75	0.1 C	4	2196	5	**[S19]**
CoOOH	91.8	0.2 C	4.35	1045	30	**[S20]**
rGO–VS_2	71.2	0.1 C	2.56	2509	10	**[S21]**
FeS_2/FeS	55.6	0.1 C	1.0~1.2	2149	20	**[S22]**
$LiCoO_2$	80	0.03 C	5.1	1520	7	**[S23]**
Co–$MoSe_2$/MXene	75	0.1 C	8	2658	3.5	**[S24]**
VN	80	0.1 C	4.3	1287	12	**[S25]**
GRO	65.5	0.2 C	8.4	4158	12	**[S26]**
$LiNi_{0.8}Co_{0.1}Mn_{0.1}O_2$	70	0.1 C	4.29	1740	10	**[S27]**
HDGS	32	0.1 C	/	491	20	**[S28]**
pOMS	79.7	0.2 C	4	1266	15	**[S29]**
IBGM	/	0.1 C	5.6	1133	5	**[S30]**
$La_{0.8}Sr_{0.2}MnO_3$	81	0.5 mA cm^{-2}	6.2	1779	7	**[S13]**
NPCN–1.8–1000	85	0.2 C	14.3	1898	8	[S31]
FLPT	61.5	0.03 C	4.2	3248	8	[S32]
TiO_2@VN	69.5	0.1 C	1.6	2735	15	**This work**
		0.5 mA cm^{-2}	4.2	1696	10	
		0.2 mA cm^{-2}	4.2	1633	7	
	80.3	0.5 C	1.8	1289	15	

Section SII. Supporting Figures

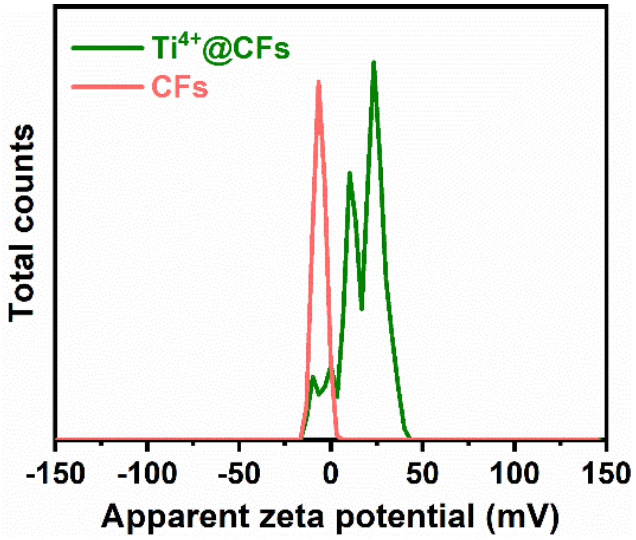

Figure S1 Zeta potentials of the CFs and Ti^{4+}@CFs dispersed in water at pH value of 4.0.

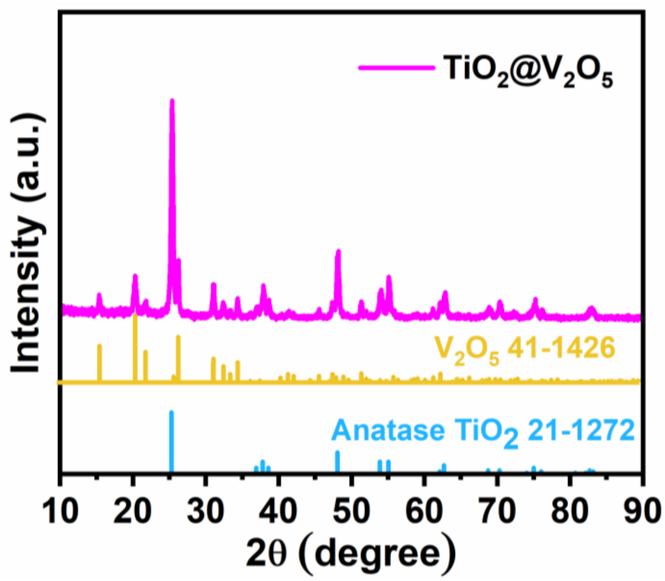

Figure S2 XRD patterns of the TiO_2@V_2O_5.

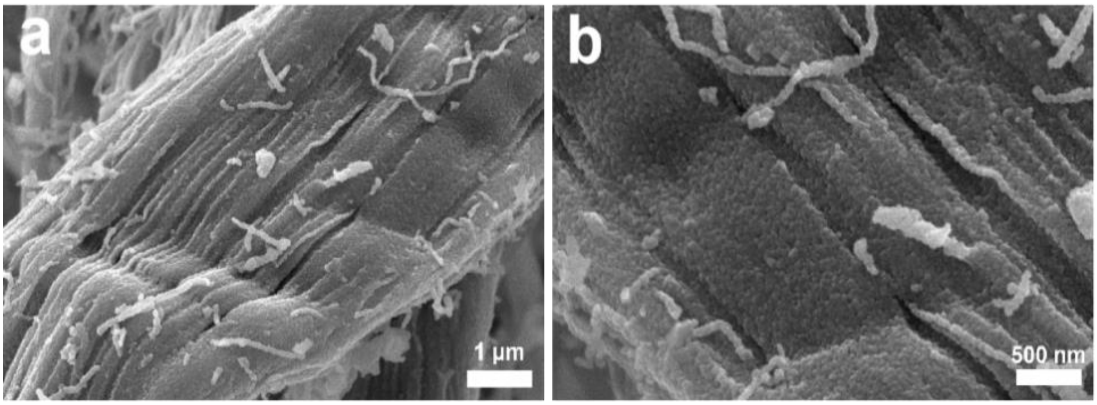

Figure S3 Typical FESEM images of the $TiO_2@V_2O_5$.

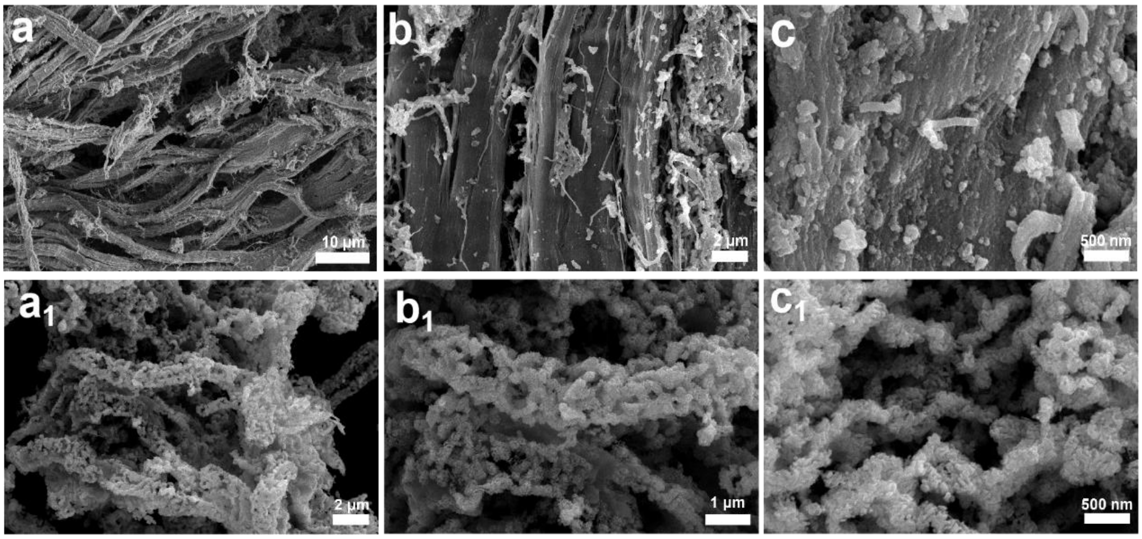

Figure S4 Typical FESEM images of the control samples: (a–c) TiO_2 and (a_1–c_1) VN.

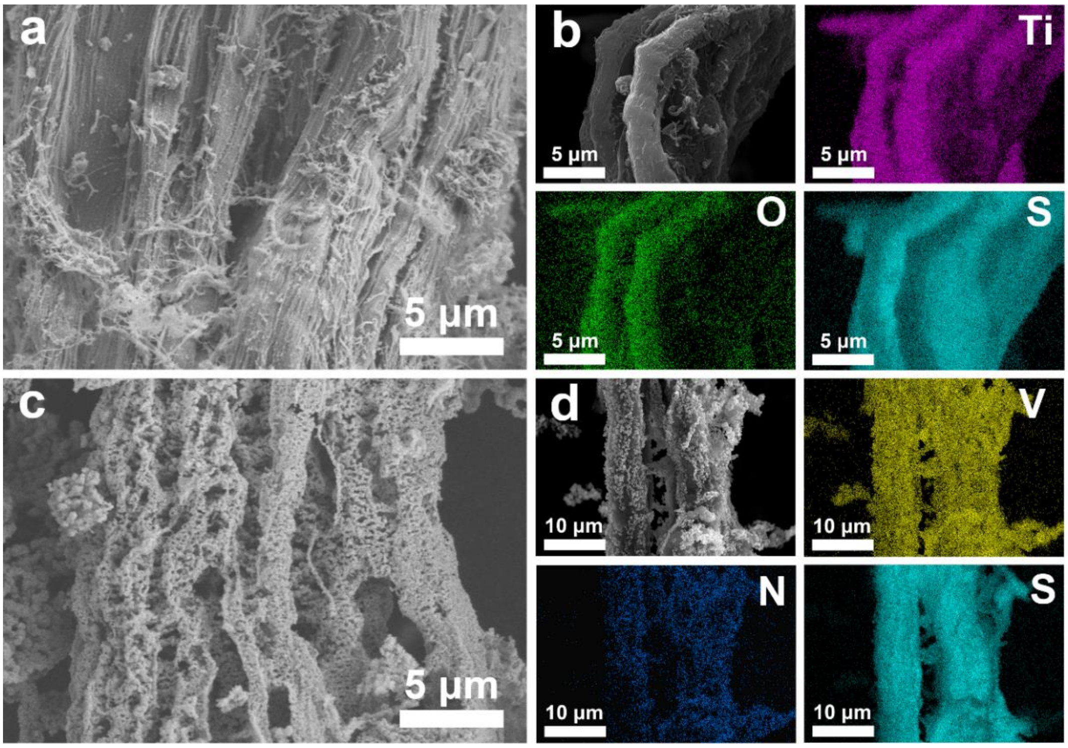

Figure S5 Typical FESEM images of the control samples after S loading: (a–b) TiO_2–S and (c–d) VN–S.

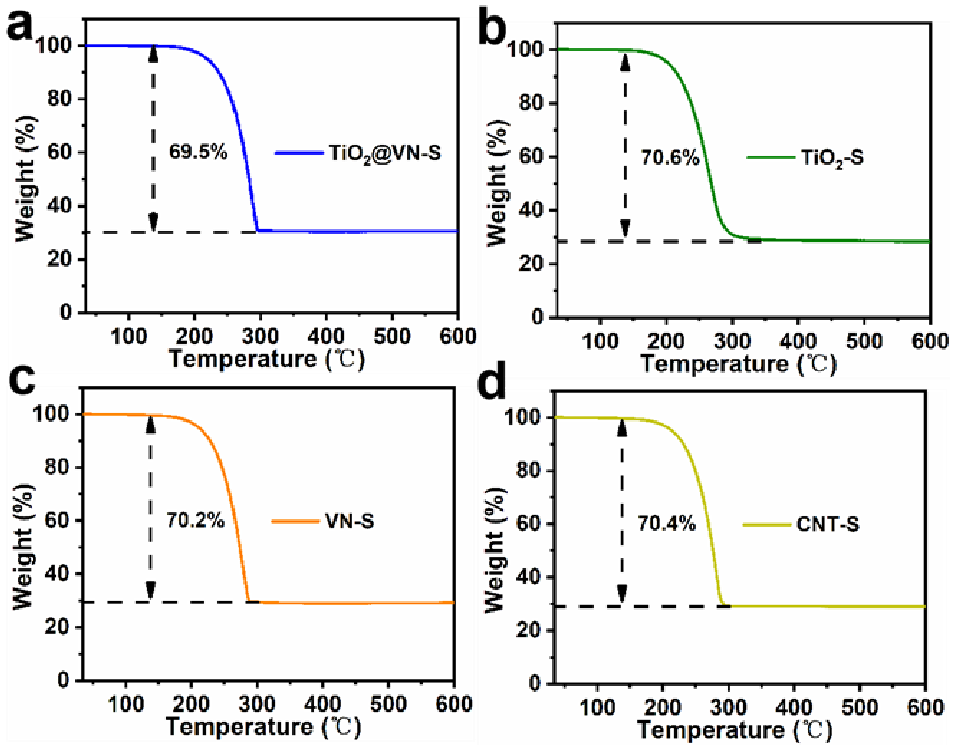

Figure S6 TGA curves of the (a) TiO_2@VN–S, (b) TiO_2–S, (c) VN–S and (d) CNT–S.

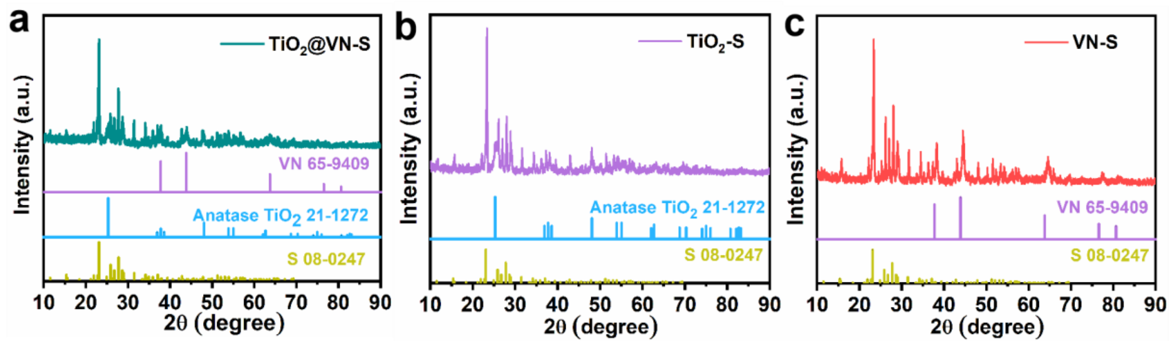

Figure S7 XRD patterns of the S–composites: (a) TiO_2@VN–S, (b) TiO_2–S and (c) VN–S.

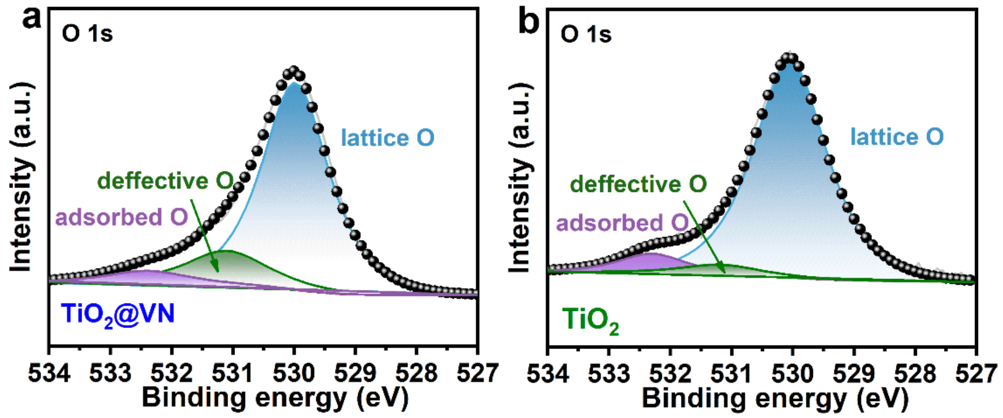

Figure S8 The high-resolution XPS spectrum of O 1s of (a) TiO_2@VN and (b) TiO_2.

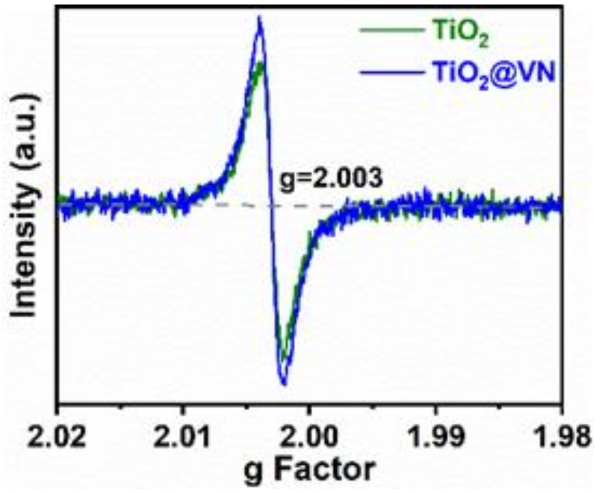

Figure S9 EPR spectrums of TiO_2 and TiO_2@VN.

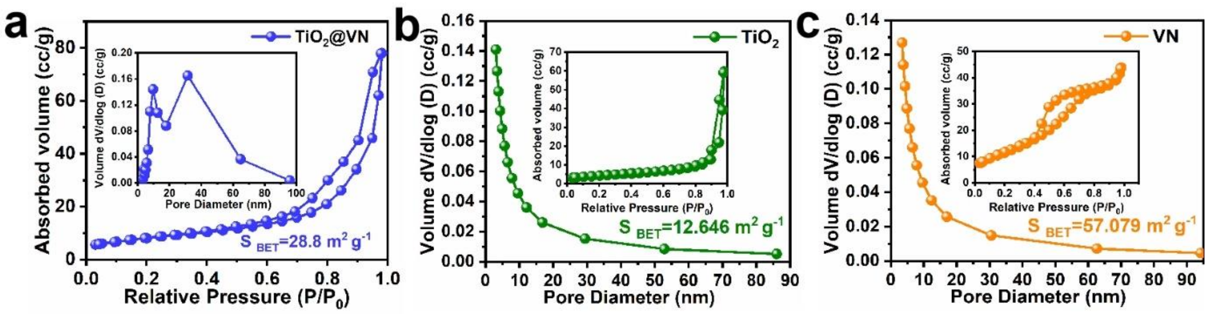

Figure S10 Nitrogen adsorption and desorption isotherms and the corresponding pore size distribution plots of the (a) TiO_2@VN, (b) TiO_2 and (c) VN.

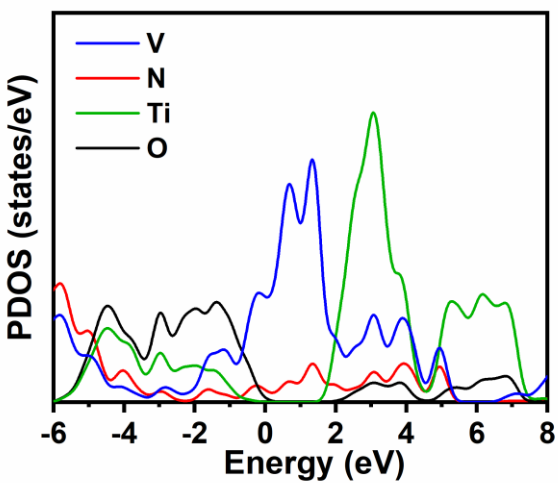

Figure S11 The partial density of states (PDOS) of TiO_2@VN heterostructure.

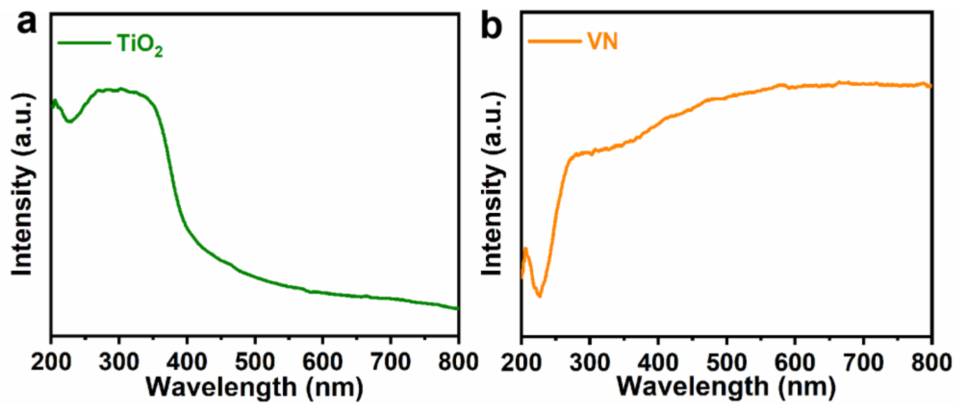

Figure S12 UV-vis DRS spectra of the (a) TiO_2 and (b) VN.

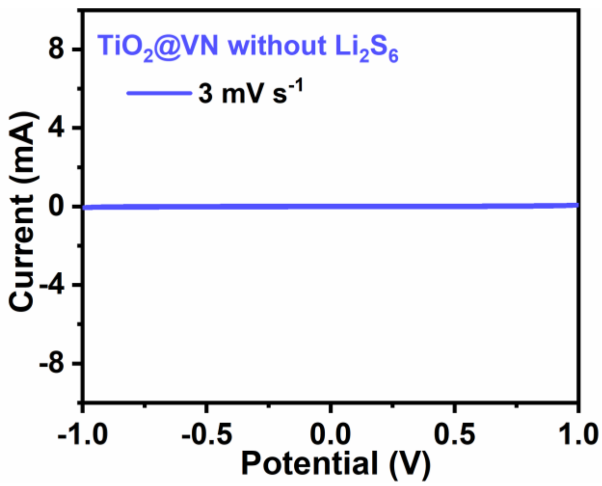

Figure S13 CV curve for the symmetrical cell of TiO_2@VN without Li_2S_6.

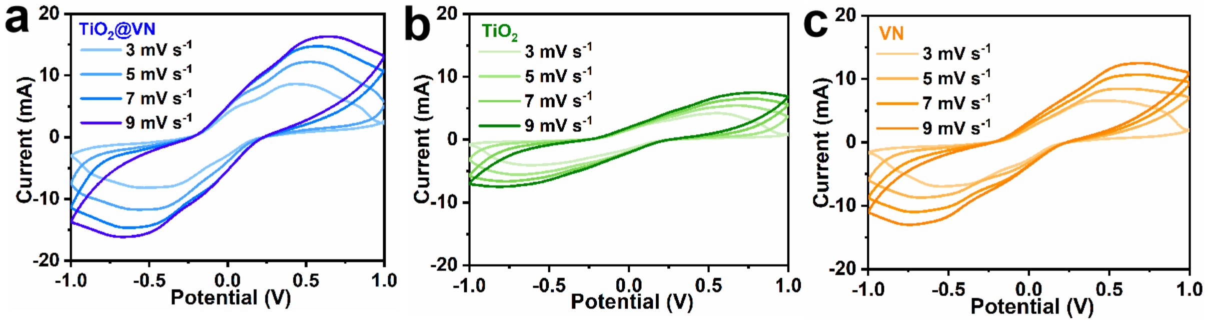

Figure S14 CV curves for the symmetrical cells of the (a) TiO_2@VN, (b) TiO_2 and (c) VN at different scan rates.

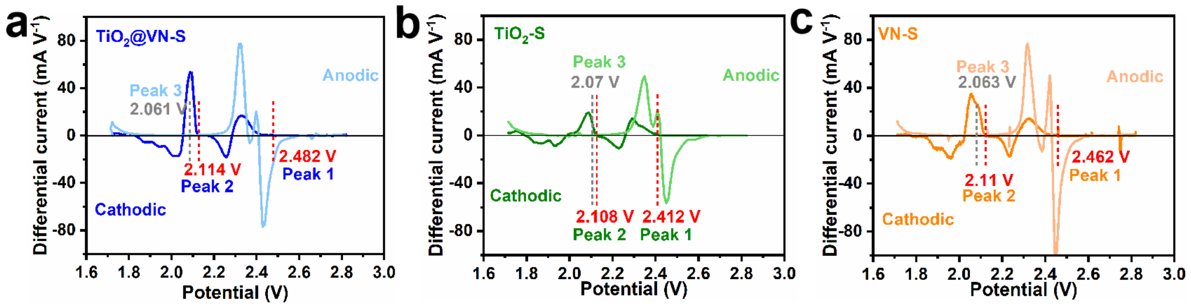

Figure S15 Differential CV curves of the (a) TiO_2@VN–S, (b) TiO_2–S and (c) VN–S at scan rate of 0.1 mV s^{-1}.

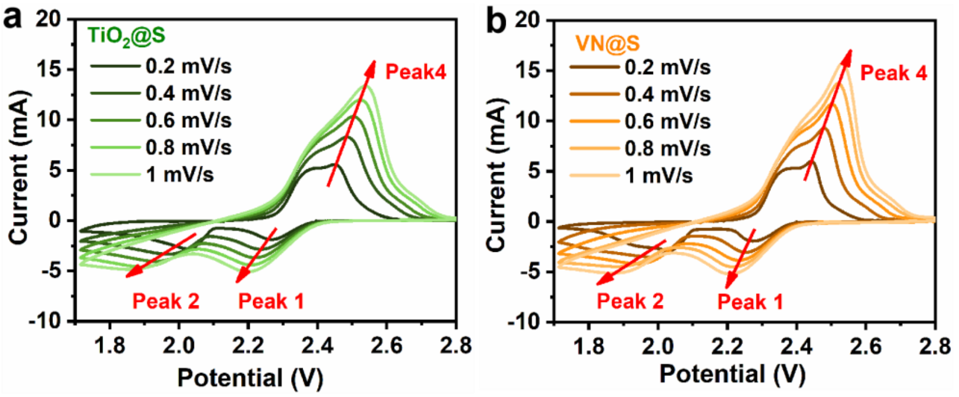

Figure S16 CV curves of the (a) TiO_2−S and (b) VN−S at different scan rates.

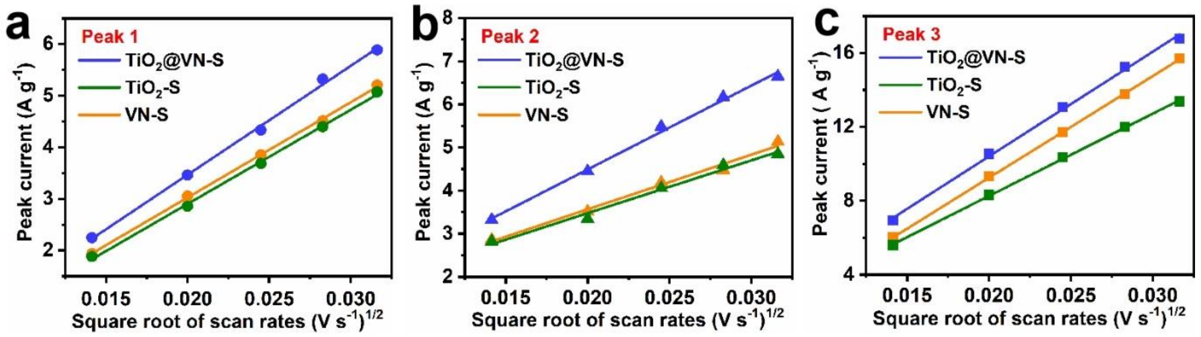

Figure S17 CV peak current versus the square root of the scan rates for the (a) first cathodic reduction process, (b) second cathodic reduction process and (c) anodic oxidation process for TiO_2@VN−S, TiO_2−S and VN−S.

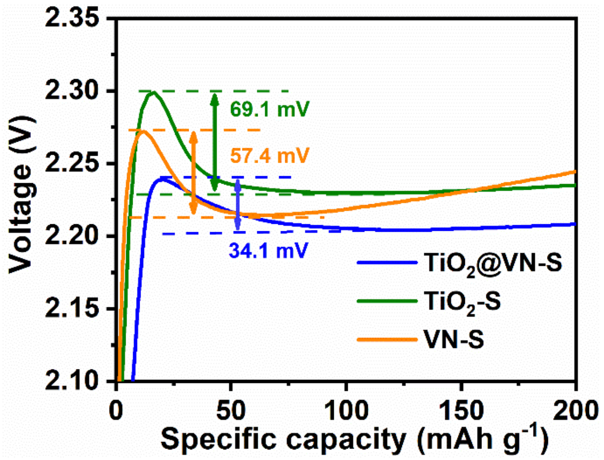

Figure S18 Corresponding enlarged part of the galvanostatic charge curves of TiO_2@VN-S, TiO_2–S, and VN–S at 0.2 C.

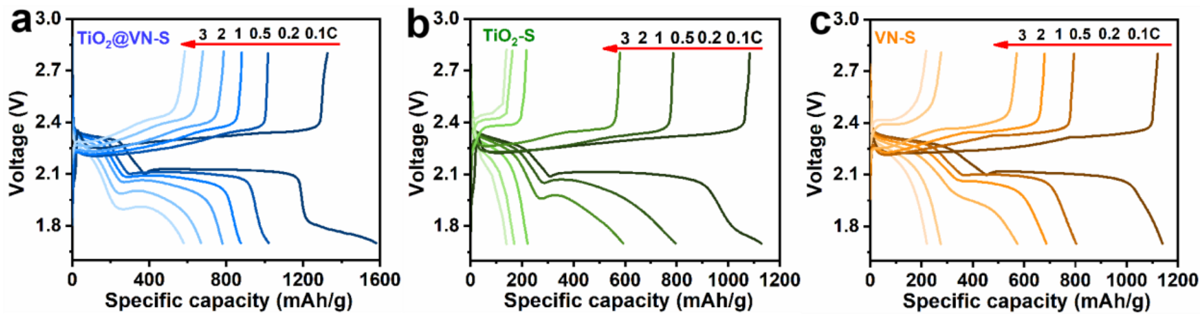

Figure S19 Typical galvanostatic discharge/charge profiles of the (a) TiO_2@VN–S, (b) TiO_2–S and (c) VN–S.

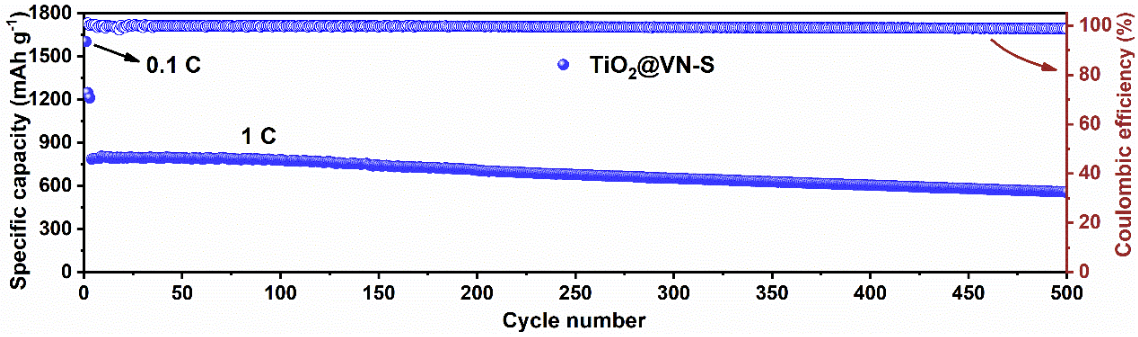

Figure S20 Cycling performance of the TiO_2@VN–S at 1 C.

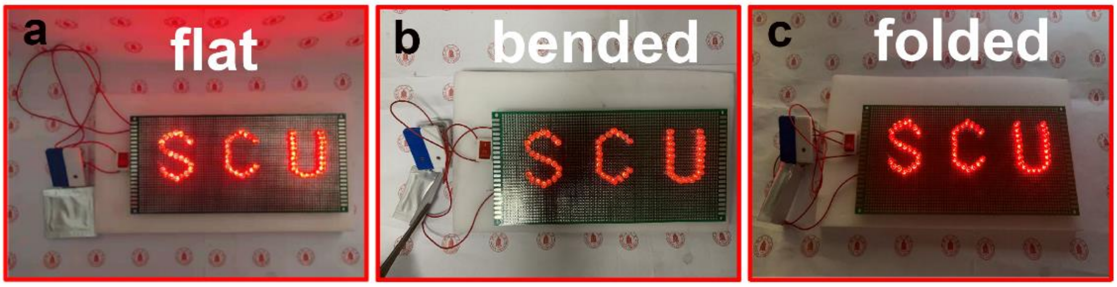

Figure S21 Digital photographs of a Li–S pouch cell in various bent states to continuously light up a “SCU” device containing 60 red light-emitting diodes (LEDs).

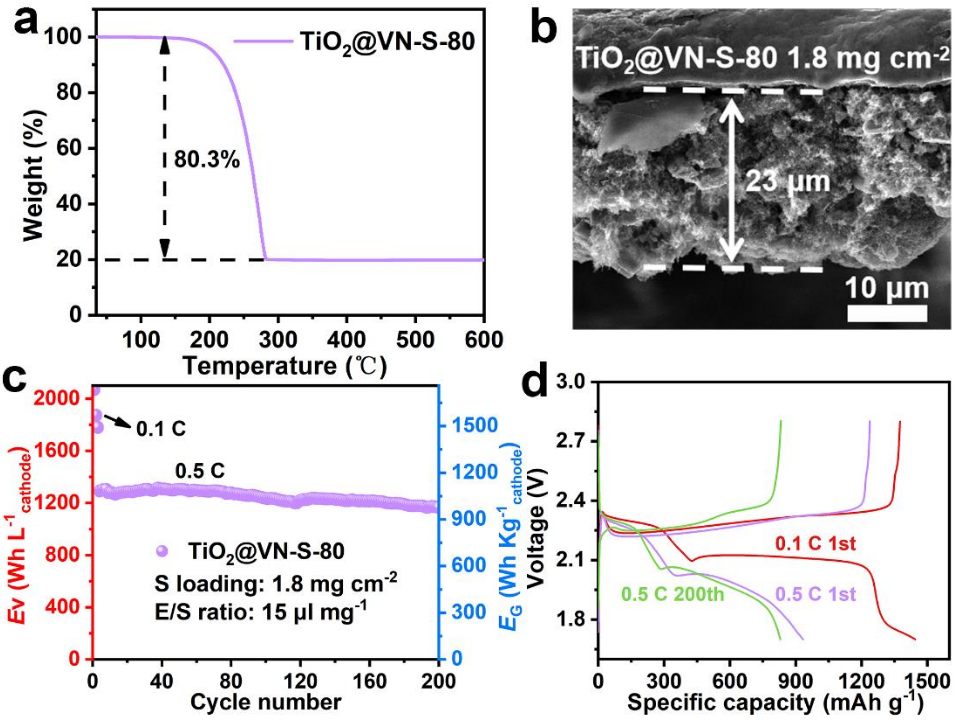

Figure S22 (a) TGA curve of the TiO_2@VN–S–80. (b) Cross-section FESEM images of the TiO_2@VN–S–80 cathode at sulfur loading of 1.8 mg cm^{-2}. (c) Cycling performance of the TiO_2@VN–S–80 cathode. (d) Typical galvanostatic discharge/charge profiles of the TiO_2@VN–S–80.

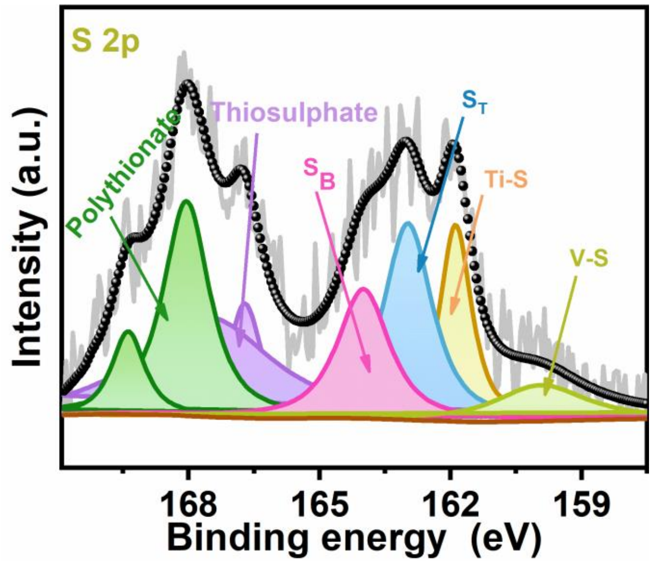

Figure S23 High-resolution XPS spectra of S 2p for the TiO_2@VN after interacting with Li_2S_6.

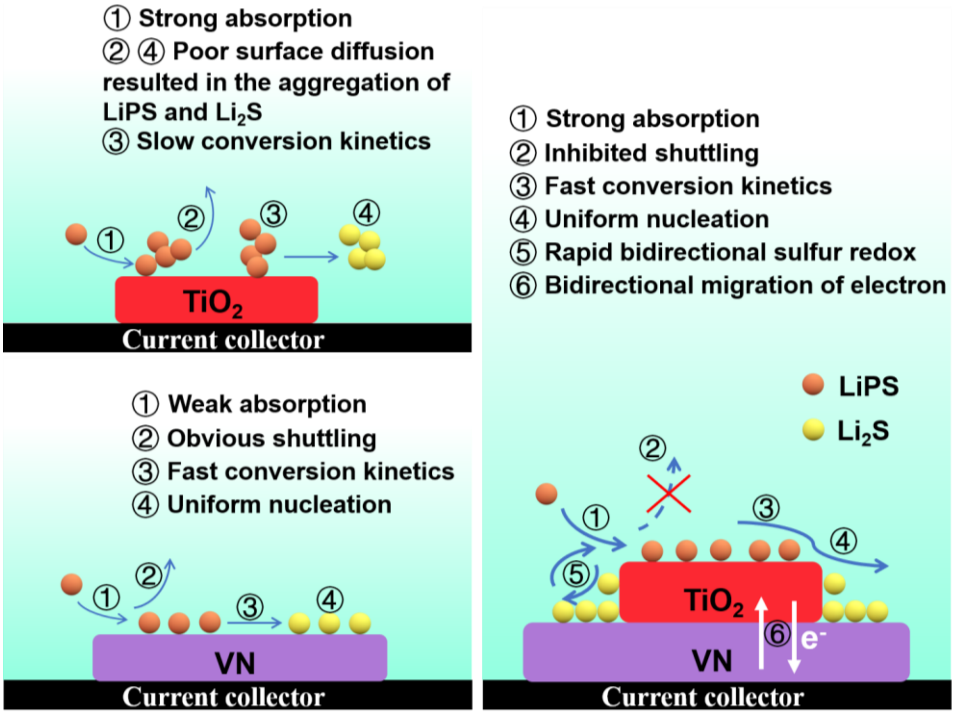

Figure S24 Schematic illustration of the role of TiO_2, VN and TiO_2@VN in tuning S chemistry.

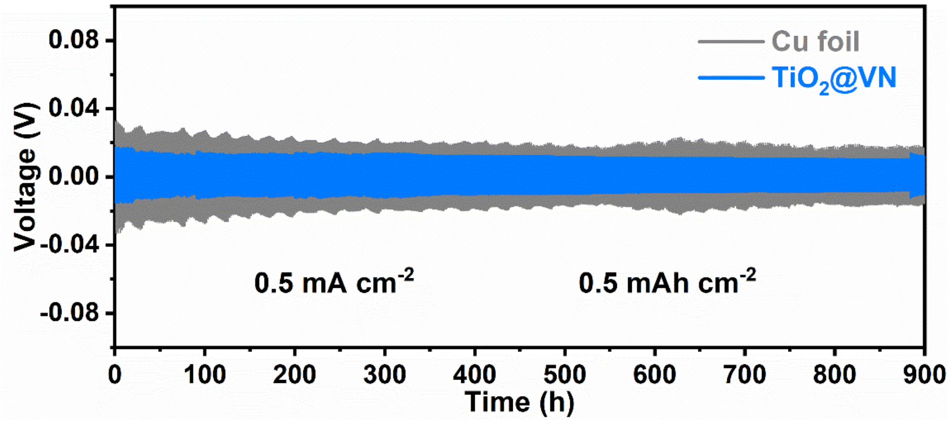

Figure S25 Galvanostatic cycling of Li|Cu–Li and Li|TiO_2@VN–Li symmetric cells at 0.5 mA cm^{-2} for 0.5 mAh cm^{-2} with pre-deposited Li of 3 mA h cm $^{-2}$.

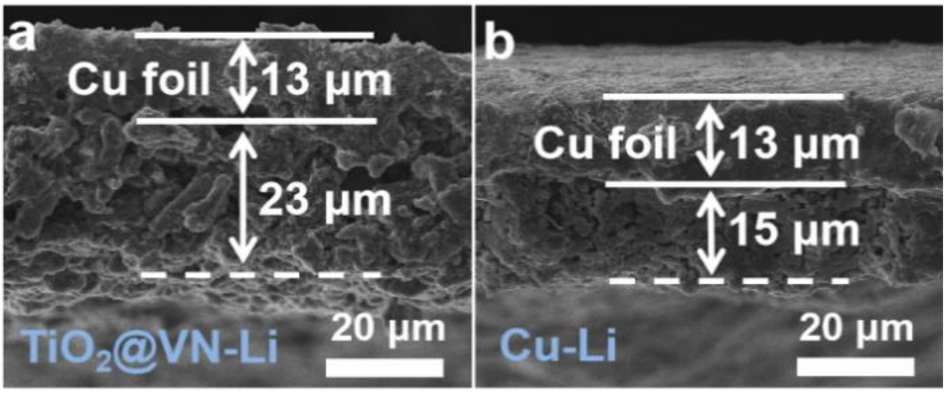

Figure S26 Cross-section SEM images of (a) TiO_2@VN–Li and (b) Cu–Li electrodes with pre-plating 4 mAh cm^{-2} of Li.

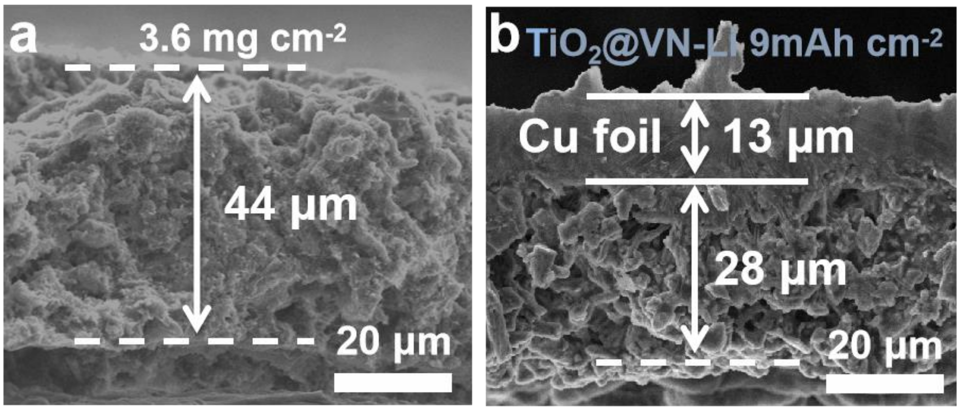

Figure S27 Cross-section SEM images of (a) TiO_2@VN–S at sulfur loading of 3.6 mg cm^{-2} and (b) TiO_2@VN–Li electrodes with pre-plating 9 mAh cm^{-2} of Li.

Reference

[S1] Y. Wei, B. Wang, Y. Zhang, M. Zhang, Q. Wang, Y. Zhang, H. Wu, Adv. Funct. Mater. **2021**, 31, 2006033.

[S2] Y. Wei, Y. Wang, X. Zhang, B. Wang, Q. Wang, N. Wu, Y. Zhang, H. Wu, ACS Appl. Mater. Interfaces **2020**, 12, 35058-35070.

[S3] Y. Wei, H. Chen, H. Jiang, B. Wang, H. Liu, Y. Zhang, H. Wu, ACS Sustain. Chem. Eng. **2019**, 7, 7823-7832.

[S4] C. Wang, H. Lu, Z. Mao, C. Yan, G. Shen, X. Wang, Adv. Funct. Mater. **2020**, 30, 2000556.

[S5] G. Kresse, J. Hafner, Phys. Rev. B **1993**, 47, 558-561.

[S6] J. Iniguez, D. Vanderbilt, L. Bellaiche, Phys. Rev. B **2003**, 67, 224107.

[S7] B. Hammer, L.B. Hansen, J.K. Norskov, Phys. Rev. B **1999**, 59, 7413-7421.

[S8] R. Asahi, W. Mannstadt, A.J. Freeman, Phys. Rev. B **1999**, 59, 7486-7492.

[S9] J.P. Perdew, K. Burke, M. Ernzerhof, Phys. Rev. Lett. **1996**, 77, 3865-3868.

[S10] J.P. Perdew, A. Ruzsinszky, G.I. Csonka, O.A. Vydrov, G.E. Scuseria, L.A. Constantin, X. Zhou, K. Burke, Phys. Rev. Lett. **2008**, 100, 136406.

[S11] P.E. Blochl, Phys. Rev. B **1994**, 50, 17953-17979.

[S12] G.I. Csonka, J.P. Perdew, A. Ruzsinszky, P.H.T. Philipsen, S. Lebegue, J. Paier, O.A. Vydrov, J.G. Angyan, Phys. Rev. B **2009**, 79, 155107.

[S13] Y.-T. Liu, S. Liu, G.-R. Li, T.-Y. Yan, X.-P. Gao, Adv. Sci. **2020**, 7, 1903693.

[S14] Z. Ling, Y. Lin, L. Shixiong, Z. Jiantao, L. Shutang, H. Qingquan, H. Kai, L. Xiaomin, W. Donghai, Q. Xuefeng, J. Mater. Chem. A **2019**, 7, 3618-3623.

[S15] Z. Xiao, Z. Li, P. Li, X. Meng, R. Wang, ACS Nano **2019**, 13, 3608-3617.

[S16] X. Yu, J. Deng, R. Lv, Z.-H. Huang, B. Li, F. Kang, Energy Stor. Mater. **2019**, 20, 14-23.

[S17] W. Xue, Z. Shi, L. Suo, C. Wang, Z. Wang, H. Wang, K.P. So, A. Maurano, D. Yu, Y. Chen, L. Qie, Z. Zhu, G. Xu, J. Kong, J. Li, Nature Energy **2019**, 4, 374-382.

[S18] Z. Zhang, D.-H. Wu, Z. Zhou, G.-R. Li, S. Liu, X.-P. Gao, Sci. China Mater. **2019**, 62, 74-86.

[S19] Y.-T. Liu, D.-D. Han, L. Wang, G.-R. Li, S. Liu, X.-P. Gao, Adv. Energy Mater.

2019, 9, 1803477.

[S20] Z.-Y. Wang, L. Wang, S. Liu, G.-R. Li, X.-P. Gao, Adv. Funct. Mater. **2019**, 29, 1901051.

[S21] Z. Cheng, Z. Xiao, H. Pan, S. Wang, R. Wang, Adv. Energy Mater. **2018**, 8, 1702337.

[S22] K. Xi, D. He, C. Harris, Y. Wang, C. Lai, H. Li, P.R. Coxon, S. Ding, C. Wang, R.V. Kumar, Adv. Sci. **2019**, 6, 1800815.

[S23] Y.-T. Liu, L. Wang, S. Liu, G.-R. Li, X.-P. Gao, Sci. China Mater. **2021**, 64, 1343-1354.

[S24] W. Wang, L. Huai, S. Wu, J. Shan, J. Zhu, Z. Liu, L. Yue, Y. Li, ACS Nano **2021**, 15, 11619-11633.

[S25] R. Liu, W. Liu, Y. Bu, W. Yang, C. Wang, C. Priest, Z. Liu, Y. Wang, J. Chen, Y. Wang, J. Cheng, X. Lin, X. Feng, G. Wu, Y. Ma, W. Huang, ACS Nano **2020**, 14, 17308-17320.

[S26] Z. Xiao, Z. Li, P. Li, X. Meng, R. Wang, Nano Energy **2020**, 70, 104522.

[S27] L. Wang, Y.-H. Song, B.-H. Zhang, Y.-T. Liu, Z.-Y. Wang, G.-R. Li, S. Liu, X.-P. Gao, ACS Appl. Mater. Interfaces **2020**, 12, 5909-5919.

[S28] C. Zhang, D.-H. Liu, W. Lv, D.-W. Wang, W. Wei, G.-M. Zhou, S. Wang, F. Li, B.-H. Li, F. Kang, Q.-H. Yang, Nanoscale **2015**, 7, 5592-5597.

[S29] L. Byong-June, K. Tong-Hyun, L. Ha-Young, J.S. Samdani, J. Yongju, Z. Chunfei, Y. Zhou, X. Gui-Liang, C. Lei, B. Seoungwoo, L. Yong Min, K. Amine, Y. Jong-Sung, Adv. Energy Mater. **2020**, 10, 1903934.

[S30] H. Li, Y. Tao, C. Zhang, D. Liu, J. Luo, W. Fan, Y. Xu, Y. Li, C. You, Z.-Z. Pan, M. Ye, Z. Chen, Z. Dong, D.-W. Wang, F. Kang, J. Lu, Q.-H. Yang, Adv. Energy Mater. **2018**, 8, 1703438.

[S31] Z. Xiao, Z. Yang, Z. Li, P. Li, R. Wang, ACS Nano. **2019**, 13, 3404-3412.

[S32] H. Pan, Z. Cheng, J. Chen, R. Wang, X. Li, Energy Stor. Mater. **2020**, 27, 435-442.
